# Effect of different end-capped donor moieties on non-fullerenes based non-covalently fused-ring derivatives for achieving high-performance NLO properties

**DOI:** 10.1038/s41598-023-28118-w

**Published:** 2023-01-25

**Authors:** Muhammad Khalid, Iqra Shafiq, Umm-e-Hani, Khalid Mahmood, Riaz Hussain, Muhammad Fayyaz ur Rehman, Mohammed A. Assiri, Muhammad Imran, Muhammad Safwan Akram

**Affiliations:** 1 Institute of Chemistry, Khwaja Fareed University of Engineering & Information Technology, Rahim Yar Khan, 64200 Pakistan; 2 Centre for Theoretical and Computational Research, Khwaja Fareed University of Engineering & Information Technology, Rahim Yar Khan, 64200 Pakistan; 3 Institute of Chemical Sciences, Bahauddin Zakariya University, Multan, 60800 Pakistan; 4 Division of Science and Technology, Department of Chemistry, University of Education, Lahore, Pakistan; 5 Institute of Chemistry, University of Sargodha, Sargodha, Pakistan; 6 Department of Chemistry, Faculty of Science, King Khalid University, P.O. Box 9004, Abha, 61413 Saudi Arabia; 7 Research Center for Advanced Materials Science (RCAMS), King Khalid University, P. O. Box 9004, Abha, 61514 Saudi Arabia; 8 National Horizons Centre, Teesside University, Darlington, DL11HG UK; 9 School of Health and Life Sciences, Teesside University, Middlesbrough, TS1 3BX UK

**Keywords:** Photonic devices, Solar cells

## Abstract

A series of derivatives (**DOCD2–DOCD6**) with D–π–A configuration was designed by substituting various efficient donor moieties via the structural tailoring of **o-DOC6-2F**. Quantum-chemical approaches were used to analyze the optoelectronic properties of the designed chromophores. Particularly, M06/6-311G(d,p) functional was employed to investigate the non-linear optical (NLO) response (linear polarizability ⟨α⟩, first (*β*_*tot*_) and second ($\upgamma$_*tot*_) order hyperpolarizabilities) of the designed derivatives. A variety of analyses such as frontier molecular orbital (FMO), absorption spectra, transition density matrix (TDMs), density of states (DOS), natural bond orbital (NBO) and global reactivity parameters (GRPs) were employed to explore the optoelectronic response of aforementioned chromophores. FMO investigation revealed that **DOCD2** showed the least energy gap (1.657 eV) among all the compounds with an excellent transference of charge towards the acceptor from the donor. Further, DOS pictographs and TDMs heat maps also supported FMO results, corroborating the presence of charge separation states along with efficient charge transitions. NBO analysis showed that π-linker and donors possessed positive charges while acceptors retained negative charges confirming the D–π–A architecture of the studied compounds. The *λ*_max_ values of designed chromophores (659.070–717.875 nm) were found to have broader spectra. The GRPs were also examined utilizing energy band gaps of *E*_HOMO_ and *E*_LUMO_ for the entitled compounds. Among all the derivatives, **DOCD2** showed the highest values of *β*_*tot*_ (7.184 × 10^–27^ esu) and $\upgamma$_*tot*_ (1.676 × 10^–31^ esu), in coherence with the reduced band gap (1.657 eV), indicating future potentiality for NLO materials.

## Introduction

A molecule develops NLO properties, when its inner electrons interact with electromagnetic radiations. The basic requirements for developing NLO material include high transmittance at harmonic as well as fundamental wavelengths. Besides, it should possess an abounding laser-induced damage threshold to enable optical intensities which offer benefit of adequate power conversion efficiency. Moreover, NLO compounds possess very high stability in the visible range of wavelength in the electromagnetic spectrum. Such compounds are gaining increasing interest among electrical engineers due to their vast applications in the fiber optic communication, optoelectronics^1^, holography^2,3^, frequency doubling^4^, protection of sensor surfaces and bioimaging^5^. The strategies that improve NLO response incorporate molecules having donor–π–acceptor configurations, extending the π-electron network, designing planar octupolar molecules, employing a push–pull mechanism, twisted π-electron systems and bond length alteration. At times organic compounds can be incorporated with metallic ligands to introduce novel nonlinear optical properties^6^. Push–pull system is a good approach to tune the photophysical properties of organic semiconductor materials (OSMs)^7^. An efficient push–pull configuration consists of three basic components: (i) donor, (ii) π-linker and (iii) acceptor which make the efficient charge separation in a molecule. The HOMO–LUMO band gap is a phenomenon that is directly associated with the push–pull mechanism. A system composed of strong electron- withdrawing groups (EWGs) linked with electron donating groups (EDGs) through π-spacers causes relative lowering of the HOMO–LUMO energy gap (*E*_gap_). This decline in *E*_gap_ consequently influences the intramolecular charge transfer (ICT) that is beneficial for designing excellent NLO compounds^8^.

Over the recent decades, tremendous has research been already being undertaken to explore bulk materials with improved NLO response, including organomatellic^9^ inorganic^10^, organic–inorganic hybrids^10,11^, and organic materials^12,13^. Every type of material has its own unique properties but organic NLO substances have found better efficacy for modern solar cell applications due to their high flexibility and small value of relative permittivity that allows robust modification. These are also considered as efficient NLO materials due to their tendency to bear high damage threshold, cheaper cost and reasonably a high photoelectric coefficient^14,15^. Organic materials also retain effective push–pull configurations fabricated from strong donors, acceptors and π-linkers. These entities become favorable NLO designs because of extended conjugation in their molecular framework hence, leading to an efficient ICT^16^. Amongst several classes of NLO materials, fullerene acceptor molecules are observed with significant nonlinear outputs^17^. Like other classes, their NLO properties could be strengthened via different types of structural alterations (employing various electron donors and acceptors). Not long since, fullerenes were widely accepted as an integral part of organic solar cells^18^. They are known as electron-deficient species with a 3-D cage-like structural configuration and exhibit robust π-aromaticity. Regardless of the π-aromaticity and the huge variety among fullerene acceptors, they are being replaced by modern class of non-fullerene acceptors (NFAs). The challenge with fullerene-based compounds is prohibited optical transitions owing to their high symmetry hindering their capability of photon absorption in the UV–visible region^19^. The non-fullerene OSCs expect to be cost-effective, lightweight and have good manufacturability, structural planarity and better stability than fullerenes^20–22^.

Computational investigations have become a credible technique for solving chemical problems appertained to molecular structures and configurations. They play a crucial role in identifying the properties regarding the chemical framework of molecules under investigation^1^. Considering these facts, we have presented a nonlinear data analysis for a non-fullerene synthesized acceptor molecule from the literature termed as **o-DOC6-2F**. The synthetic procedure for the selected NF compound has been reported by Hou et al*.*^23^ Herein, we have formulated innovative designs of D–π–A nature via structural fabrication of the reference (A–π–A). This structural modification is done utilizing a few exceptionally strong donors to produce a push–pull π-conjugated framework thus achieving high NLO responses of these compounds^24^. For a detailed understanding, complete theoretical study of compounds is executed utilizing one of the emerging computational approach entitled, density functional theory (DFT) and also time-dependent DFT. The acceptance of DFT-based findings is increasing as they match well with the experimental results. For this purpose, Minnesota functionals have been most commonly employed with a suitable basis sets as here, M06 functional was used along with 6–311 G(d,p) basis sets. The calculations involved geometrical optimization, UV–Vis, FMO, GRPs, NBO, TDM, DOS and NLO analyses. These unique NFAs-based non-linear optical materials would be a great addition to developing high-tech compounds in the future.

### Computational procedure

The molecular geometries were optimized at ground state S_0_ without any symmetry restrictions using the M06^25^ functional along with 6-311G(d,p) basis set to perform all the computational calculations. The software employed for this purpose was Gaussian 09^26^ system from the lab facilities provided by Dr. Ataualpa Albert Carmo Braga. The FMOs diagrams were achieved using Avogadro software^27^ which helped to show the highest occupied and the lowest unoccupied molecular orbitals along with their energies. Another important analysis was the NBO study for determining the stabilization pattern of the studied compounds which was performed with NBO software package 3.1^28^^,^^29^. The UV–Vis spectral analysis was performed using TD-DFT method at an aforesaid level employing the Gauss Sum^30^ and Origin^31^ software programs. NLO properties of entitled chromophores were also examined at the aforementioned functional. The Eq. (1) was used for *β*_*tot*_.

1 $$\begin{aligned} & \beta_{tot} = \, \left[ {\left( {\beta_{xxx} + \beta_{xyy} + \beta_{xzz} } \right)^{{2}} + \, \left( {\beta_{yyy} + \beta_{xxy} + \beta_{yzz} } \right)^{{2}} + \, \left( {\beta_{zzz} + \beta_{xxz} + \beta_{yyz} } \right)^{{2}} } \right]^{{{1}/{2}}} \\ & {\text{Where}},\,\beta_{x} = \beta_{xxx + } \beta_{xyy + } \beta_{xzz} ,\beta_{y} = \beta_{yxx + } \beta_{yyy + } \beta_{yzz} \,{\text{and}}\,\beta_{z} = \beta_{zxx + } \beta_{zyy + } \beta_{zzz} \\ \end{aligned}$$

The other nonlinear parameters like linear polarizability < α >^32^ and second-hyper polarizability $\upgamma$_*tot*_ were also calculated with the help of the following Eqs. (2) and (3).

2 $$\left\langle \upalpha \right\rangle = {1}/{3}\left( {\upalpha _{{{\text{xx}}}} + \upalpha _{{{\text{yy}}}} + \upalpha _{{{\text{zz}}}} } \right)$$

3 $${\upgamma }_{{{\text{tot}}}} = \sqrt {{\upgamma }_{x }^{2} + {\upgamma }_{y}^{2} + {\upgamma }_{z}^{2} }$$

where $$\gamma_{i} = \frac{1}{15 }\sum\nolimits_{j} {(\gamma_{ijji} + \gamma_{ijij} + \gamma_{iijj} )} \quad i,j = \left\{ {x, y, z} \right\}$$

## Results and discussion

**Figure 1:**
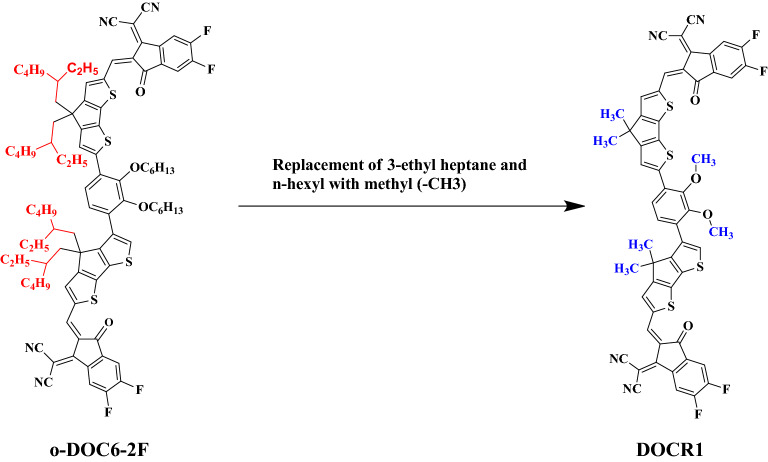
Side-chain modification of the parent molecule (**o-DOC6-2F)**^28^ to convert into a reference (**DOCR1**) molecule. These structures are drawn with the help of ChemDraw software (https://chemistrydocs.com/chemdraw-pro-8-0/).

In this paper, a non-covalently fused closed-chain electron acceptor is taken as a parent molecule that belongs to a class of NF. The IUPAC name of the parent compound is 2-((Z)-2-((6-(4-(6-((Z)-(1-(dicyanomethylene)-5,6-difluoro-3-oxo-1H-inden-2(3H)-ylidene)methyl)-4,4-bis(2-ethylhexyl)-4H-cyclopenta[1,2-b:5,4-b′]dithiophen-2-yl)-2,3-bis(hexyloxy)phenyl)-4-(5,7-diethylundecan-6-yl)-4H-cyclopenta[1,2-b:5,4-b′]dithiophen-2-yl)methylene)-5,6-difluoro-3-oxo-2,3-dihydro-1H-inden-1-ylidene)malononitrile abbreviated as **o-DOC6-2F**^28^. It is simplified by a few side-chain modifications (replacing long-chain groups with a methyl group to reduce the computational cost) into a new molecule which is taken as a reference compound and coded as **DOCR1** (Fig. 1). The **DOCR1** possess an A–π–A configuration with the same acceptor (A) present at both ends, which are named as 2-(5,6-difluoro-2-methylene-3-oxo-2,3-dihydro-1H-inden-1-ylidene)malononitrile while, the π-spacer is named as 2-(4-(4,4-dimethyl-4H-cyclopenta[1,2-b:5,4-b′]dithiophen-3-yl)-2,3-dimethoxyphenyl)-4,4-dimethyl-4H-cyclopenta[1,2-b:5,4-b′]dithiophene. It is chemically tailored using some prominent donor groups leading to some unique D–π–A derivatives (**DOCD2–DOCD6**) by replacing a terminal acceptor with axial donors. The IUPAC names of these above-mentioned compounds are presented in supplementary data along with their codes.

**Figure 2:**
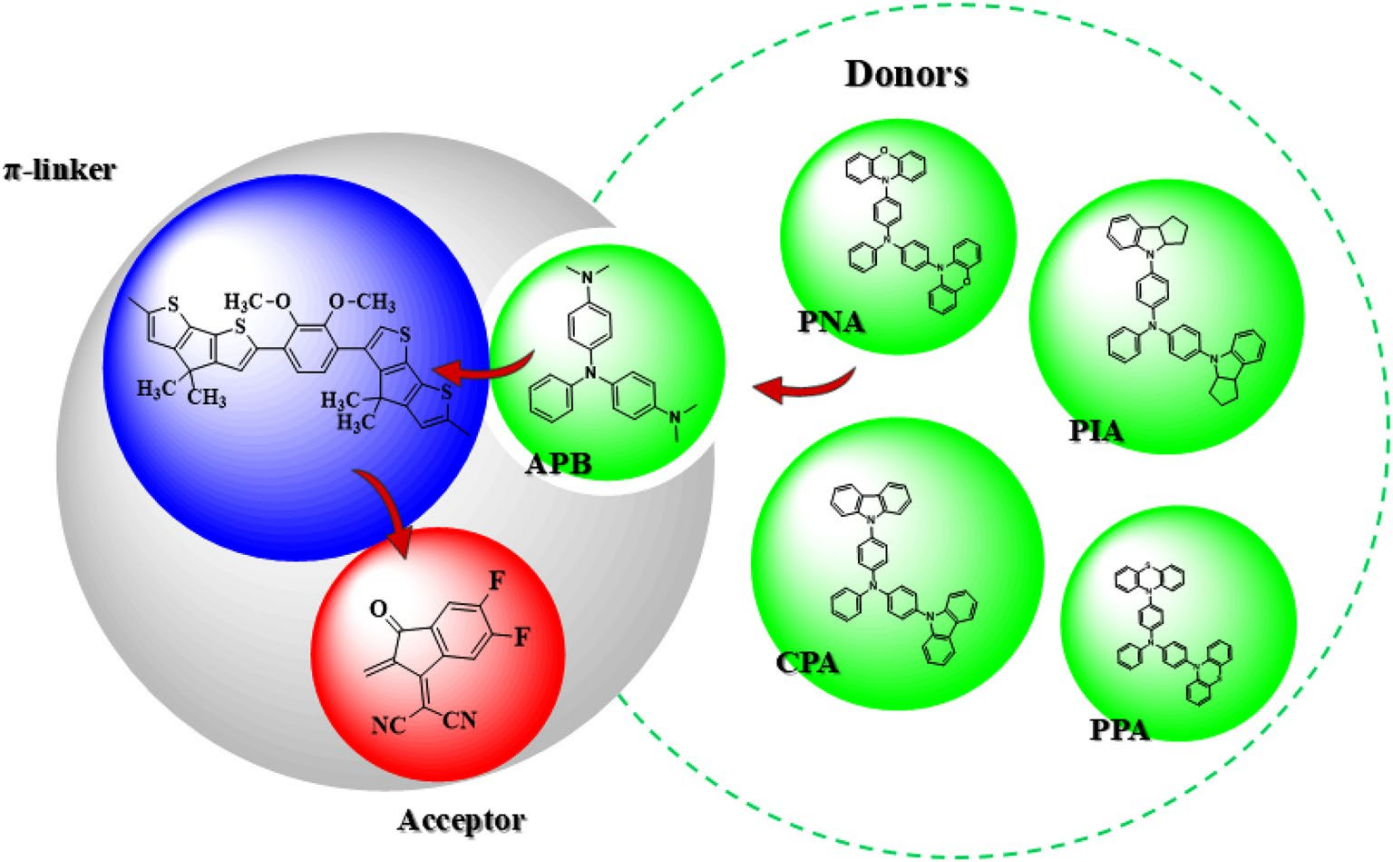
A sketch map of the designed compounds. This scheme is drawn with the help of ChemDraw software (https://chemistrydocs.com/chemdraw-pro-8-0/).

**Figure 3:**
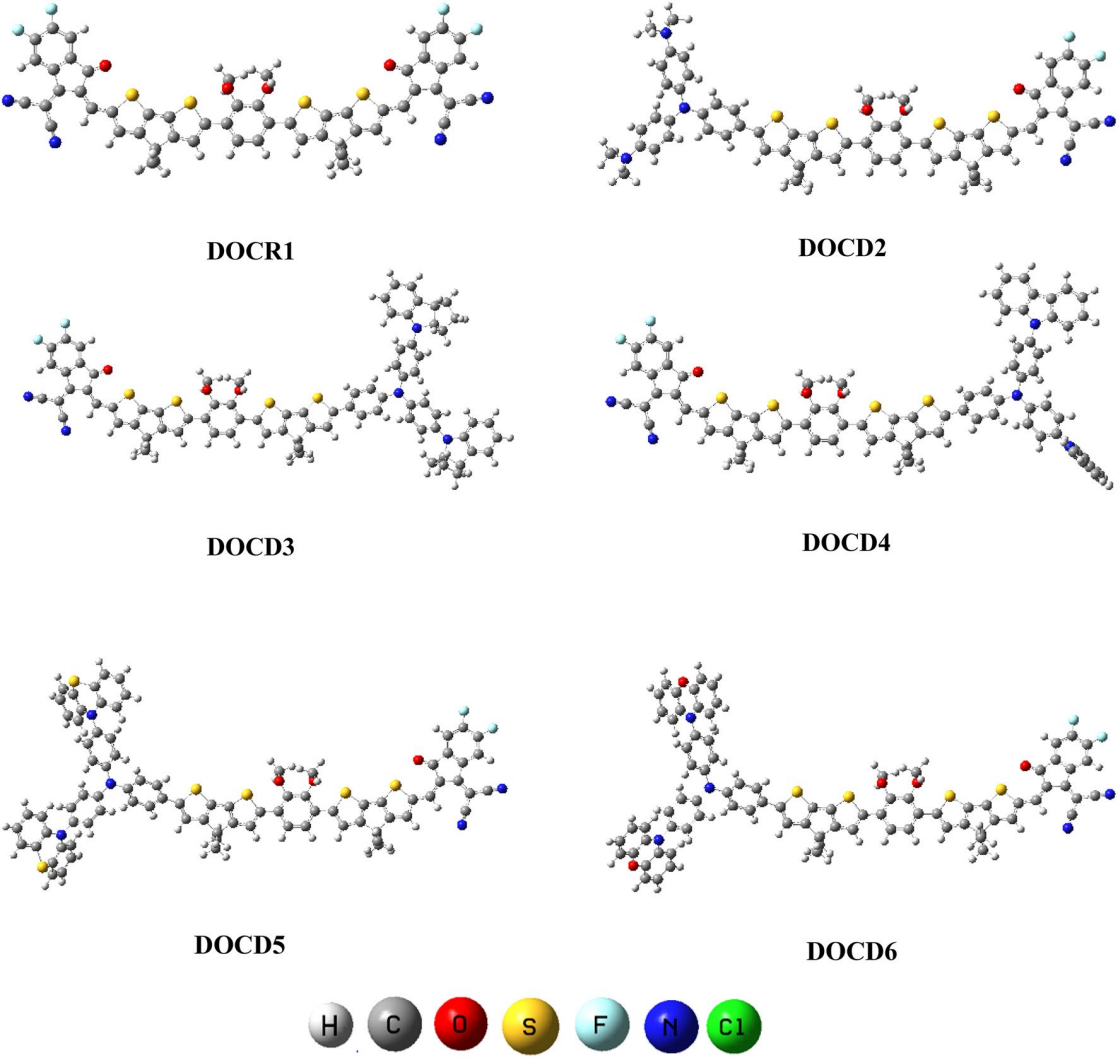
Optimized structures of **DOCR1** as well as **DOCD2**–**D6.** Figures are made with are made with the help of GaussView 5.0 and Gaussian 09 version D.01 (https://gaussian.com/g09citation/).

Figures 2, 3 and S1 represent the structural modulation of the reference compounds along with the structures of donor atoms utilized for this purpose. Following the structural designing of derivatives, they are assessed for the following parameters by using M06/6-311G(d,p) functional: (i) energy band gap (*E*_g_); (ii) UV–Vis absorption (*λ*_max_); (iii) stabilization energy (*E*^(2)^); (iv) chemical reactivity parameters like electronegativity (*X*)^33^, global softness (*σ*), ionization potential (*IP*), electron affinity (*EA*), hardness (*η*)^34^ and electrophilicity index (*ω*)^35^; (v) binding energy (*E*_b_); (vi) HOMO–LUMO contributions (DOS) and (vii) NLO properties ($\mu$_*tot*_, <α> *, β*_*tot*_ and $\upgamma$_*tot*_). The present NLO-based computational analysis would be a remarkable addition to the research field and possibly allow the organic chemists to synthesize these compounds.

### Frontier molecular orbital (FMO) analysis

The study of the electronic structure of the chromophores provided by the FMOs analysis plays a significant role in determining their non-linear optical properties^36^. The quantum orbitals entitled as HOMO and LUMO unveil charge transfer efficiency from the higher to lower levels in a molecule^37^. The HOMO is known as the electron donor orbital while, the LUMO is at a lower energy level, regarded as the electron acceptor molecular orbital^38^. The FMO energy gap is considered as a useful tool in deducing the dynamic stability and chemical reactivity of a substance^1,39–45^. Table 1 manifests the energy band gap for all the studied compounds which is obtained as the difference between HOMO and LUMO energy values (*E*_LUMO_ − *E*_HOMO_).

**Table 1:** Energies of frontier molecular orbitals of **DOCR1** and **DOCD2–DOCD6** molecules.

Compounds	*E*_HOMO_	*E*_LUMO_	Band gap
DOCR1	− 5.757	− 3.405	2.352
DOCD2	− 4.894	− 3.237	1.657
DOCD3	− 5.033	− 3.246	1.787
DOCD4	− 5.320	− 3.255	2.065
DOCD5	− 5.374	− 3.258	2.116
DOCD6	− 5.379	− 3.257	2.122

Band gap = *E*_LUMO_ − *E*_HOMO_, units in e*V.*

In order to interpret the chemical nature of a molecule, it is important to comprehend the movement of electrons from HOMO towards LUMO. The data of Table 1 indicates HOMO–LUMO values of the reference compound **DOCR1** as − 5.757 and − 3.405 eV which shows a good harmony with experimental values such as − 5.54 and − 3.85 eV^23^, respectively that indicated the suitable selection of functional for the current study. It can be clearly seen from the obtained results that the HOMOs in all derivatives (**DOCD2–DOCD6**) are present at the higher position than **DOCR1** as they possess higher energy values *i.e.* − 4.894, − 5.033, − 5.320, − 5.374, − 5.379 eV, respectively. Similarly, in the case of LUMO, all the compounds show higher LUMO energies as − 3.237, − 3.246, − 3.255, − 3.258 and − 3.257 eV for **DOCD2**, **DOCD3**, **DOCD4**, **DOCD5** and **DOCD6**, respectively. So, the LUMOs of all the derivatives lie at a much lower position along with elevated level of HOMO yielding a high probability of charge transference in compounds.

**Figure 4:**
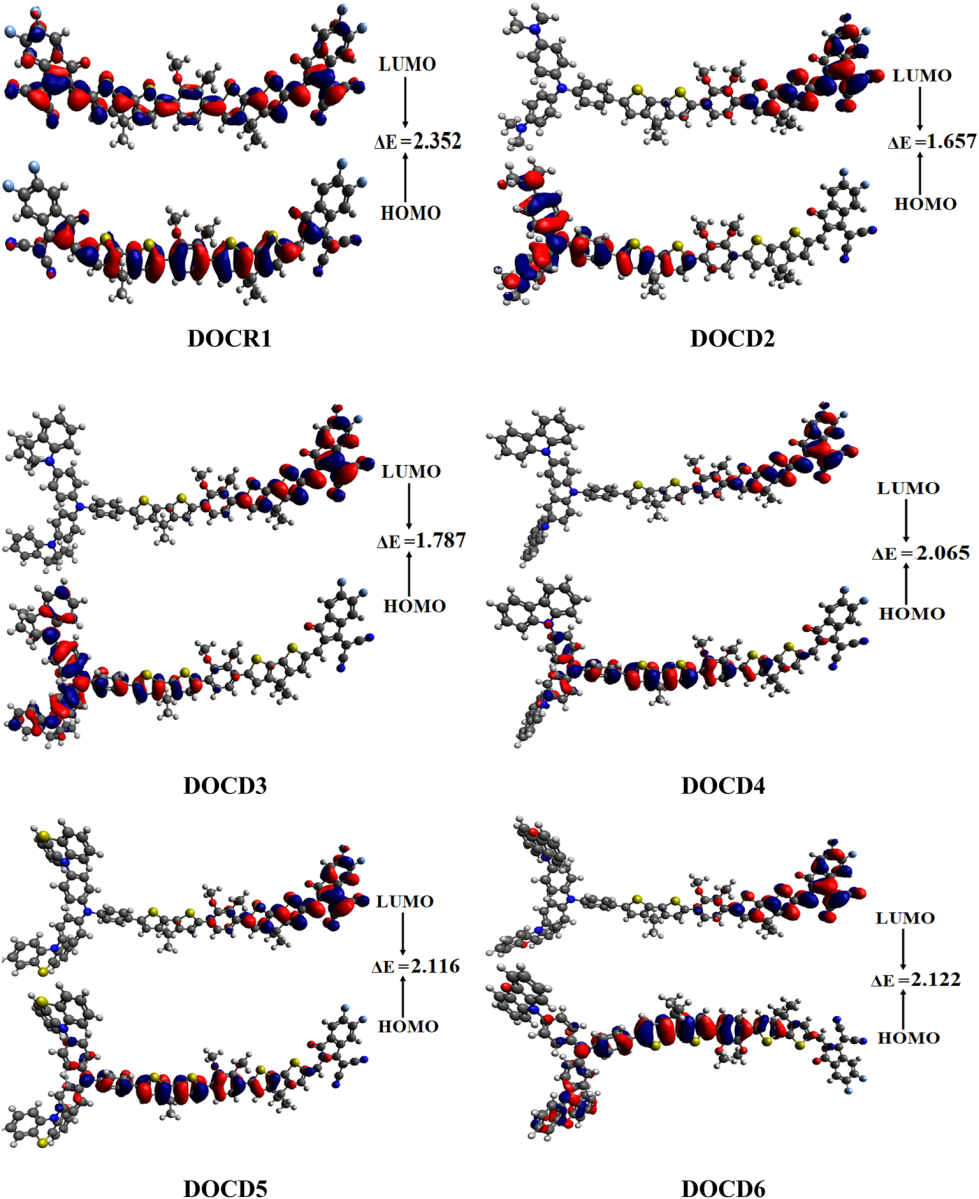
HOMO–LUMO distribution patterns for **DOCR1** and **DOCD2–DOCD6**, units in e*V.* Figures are drawn with the help of Avogadro software, Version 1.2.0. (http://avogadro.cc/). All output files of entitled compounds were accomplished by Gaussian 09 version D.01 (https://gaussian.com/g09citation/).

Figure 4 shows the pictorial demonstrations of HOMOs and LUMOs of the designed compounds. Here, the negative phase of molecular orbitals is indicated by the in red shade, while the positive phase is indicated by the blue color. The band gap can simply be used to assess the polarizable nature of compounds. In this case, a smaller band gap indicates more ICT from the electron donor towards the acceptor parts within a molecule, and such compounds have high chemical polarizability. The compound **DOCD2** has shown the lowest value of the HOMO–LUMO energy gap *i.e.* 1.657 eV, as illustrated in Table 1. This might be due to a suitable engineered donor induced in the molecule named as; *N,N*-dialkylaniline and shows reasonable electron donating tendency within **DOCD2** (Fig. 1).

The compound **DOCD3** revealed slightly higher band gap than **DOCD2** (1.787 eV) due to incorporated indoline as a donor part. Furthermore, the other compounds (**DOCD4**, **DOCD5** and **DOCD6**) also demonstrate significantly higher energy band gaps than **DOCD2** *i.e.* 2.065, 2.116 and 2.122 eV. The donor species accompanied by these derivatives are carbozole, phenothiazine and phenoxazine, respectively. The orbital energy gap in all the series of derivatives are arranged in ascending order as: **DOCD2 **˂ **DOCD3 **˂ **DOCD4 **˂ **DOCD5 **˂ **DOCD6**˂**DOCR1** (see Table 1). Concluding the above discussion, the derivative coded **DOCD2** is seemed to be the most polarizable designed molecule in the series.

Moreover, the overall results obtained are interesting meeting our expectations as all the designed derivatives have shown lower band gaps than the reference compound. It is inferred that these derivatives have a bathochromic shift as compared to the fused ring electron acceptor molecule (**DOCR1**). Further, from Fig. 4, excellent charge transference from donor to acceptor via π-bridge is done. Hence, our engineered molecules may be appealing to high-performance NLO material.

### Density of states (DOS) analysis

**Figure 5:**
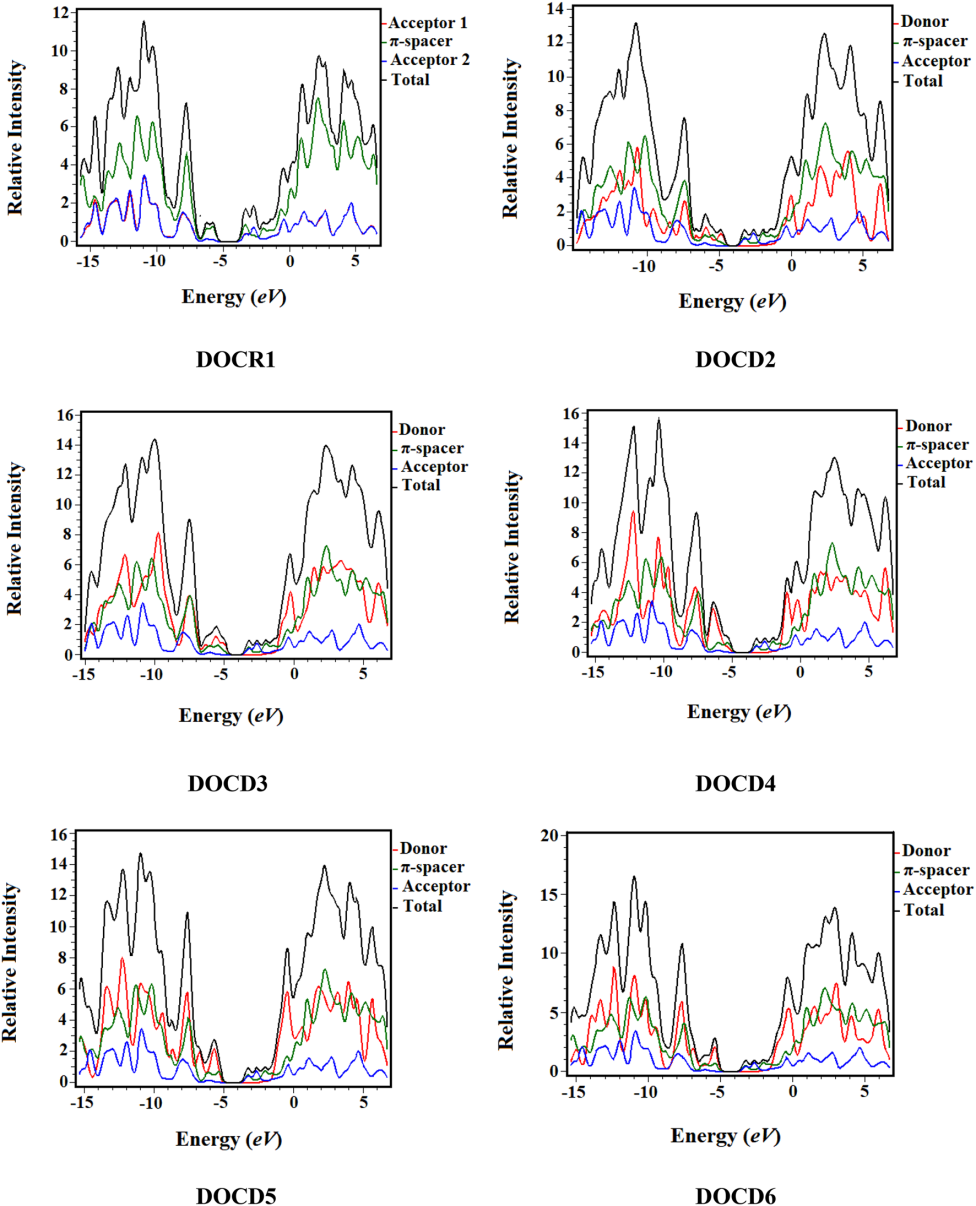
Density of states diagrams of **DOCR1** and **DOCD2–DOCD6** at M06/6-311G(d,p) level. Figure was drawn by utilizing PyMOlyze 1.1 version and output files were computed through Gaussian 09 version D.01.

The DOS plots are used for elucidating results obtained from FMO analysis upon examining the role of donor groups in the designed molecules (**DOCR1** and **DOCD2–DOCD6**). For this purpose, we divided our compounds into acceptor, donor and π-spacer, demonstrated by red, blue and green colored line graph, respectively (Fig. 5). In DOS pictographs, the HOMO represents the valence band exhibiting negative values while the positive values are depicted by the conduction band (LUMO)^46,47^. Moreover, it also displays charge density on the acceptor, donor and π-spacer fragments^48^. In **DOCR1**, the electronic charge density at HOMO and LUMO is distributed across the core unit (π-spacer). Utilization of different donor motifs alter the arrangement of electronic charge on MO that are explained through DOS percentages on HOMO and LUMO^46^. For derivatives **DOCD2–DOCD6**, the charge density for HOMO is mainly distributed over donor and significantly on the π-linker. In LUMO, it is prominently present over the the π-spacer motif and slightly on the acceptor region. The percentages of electronic distribution on the HOMO for acceptor (A) are 10.1, 0.4, 0.4, 2.4, 3.1 and 2.5% while, on LUMO they are 20.5, 45.3, 45.4, 45.6, 45.6 and 46.1% for **DOCR1** and **DOCD2–DOCD6**, respectively. For these novel compounds, the charge density is contributed by the donor (D) at HOMO is 10.1, 77.1, 76.8, 31.8, 22.4 and 32.6% and at LUMO it is 20.9, 0.2, 0.2, 0.2, 0.2 and 0.1%, respectively. DOS analysis for **DOCR1** and designed compounds **DOCD2**, **DOCD3**, **DOCD4**, **DOCD5** and **DOCD6** strongly imply charge transference from the donor towards the acceptor facilitated by the π-bridge. Hence, the tailored donor groups in these designed compounds efficiently push electrons towards the acceptor moieties creating a stronger push and pull mechanism.

### Absorption analysis

TD-DFT computations were performed via M06/6-311G(d,p) combination to comprehend the absorption spectra for the excited states of **DOCR1** and **DOCD2–DOCD6**. Data concerning charge transfer probability, configurations leading to transition and the nature of electronic transition are elucidated by the UV–Vis spectroscopy^41,49,50^. As reported by the Franck–condon principle, vertical excitation is associated with the highest absorption peak (*λ*_max_) in the spectrum. From the aforementioned computations, permissible singlet–singlet six lowest transitions are analyzed utilizing TD-DFT study^51^. Besides, effects on molecular spectra of the computed compounds by donor and acceptor moieties are also evaluated. The *λ*_max_ of our investigated compounds reveals their absorbance in the visible region of the electromagnetic spectrum as shown in Table 2.

**Table 2:** Maximum absorption wavelengths (*λ*_max_), transition energy (*eV*), oscillator strengths (*f*_os_) and transition types of computed compounds.

Compounds	*λ*_*max*_(*nm*)	*E*(*eV*)	*f*_*os*_	Major MO attributes (%)
DOCR1	683.447	1.814	3.394	H → L (92%)
DOCD2	667.730	1.857	1.552	H − 1 → L (84%)
DOCD3	659.070	1.881	1.309	H − 1 → L (81%)
DOCD4	717.875	1.727	1.779	H → L (89%)
DOCD5	706.382	1.755	1.881	H → L (90%)
DOCD6	700.792	1.769	1.789	H → L (81%)

*MO* molecular orbital; *H* HOMO, *L* LUMO.

**Figure 6:**
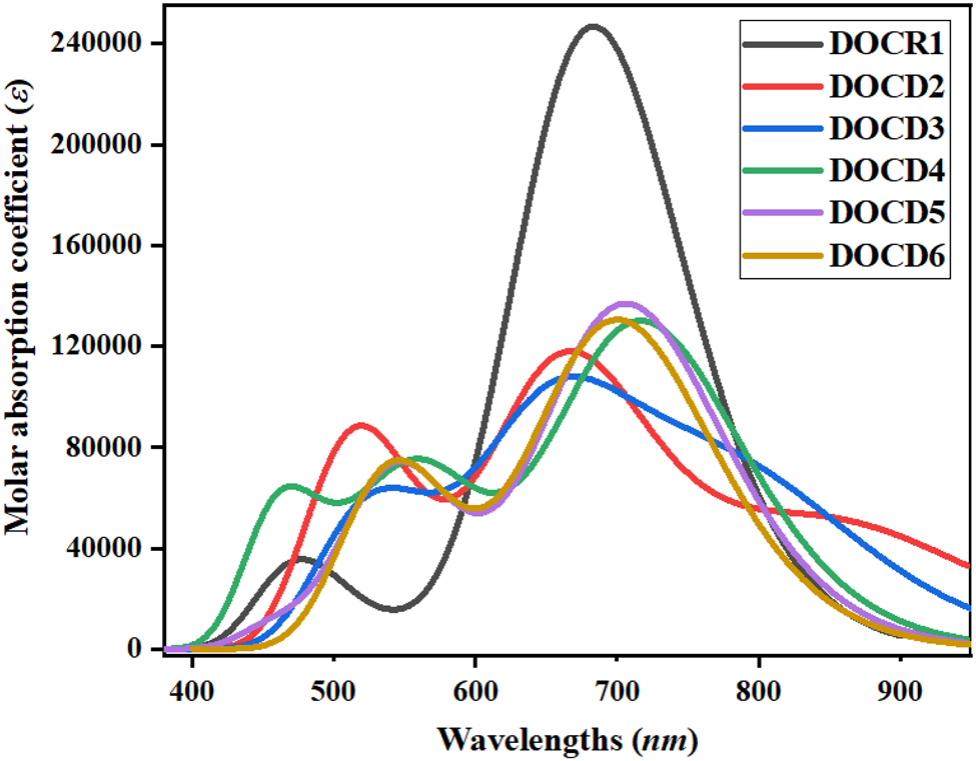
UV–Visible absorption spectra of **DOCR1** and **DOCD2–DOCD6.** These graphs were drawn by utilizing the Origin Pro 8.5 version.

Figure 6 represents the simulated absorption spectra of the studied derivatives with an absorption range of 700.792 to 717.88 nm (**DOCD4**–**DOCD6**) higher than the *λ*_max_ of **DOCR1** *i.e.* 683.45 nm. The absorption spectrum of reference chromophore (*λ*_max=_683.447 nm) exhibited good harmony with experimental results (*λ*_max=_683 nm) that supports the suitable selection of DFT functional^23^. However, derivatives **DOCD2** and **DOCD3** reveal 1.023 and 1.036 times less absorption value than that of **DOCR1** (667.730 and 659.070 nm, respectively). The *λ*_max_ values (Table 2) are greatly influenced by donor moieties in the structure owing to the push–pull configuration in the proposed NLO compounds. The highest absorption peak of reference (**DOCR1**) is 683.447 nm with 1.814 eV transition energy and *f*_*os*_ of 3.394, revealing 92% contributions of molecular orbitals from HOMO to LUMO. On introducing the donor (N-(4-(dimethylamino)phenyl)-N,N-dimethyl-N-phenylbenzene-1,4-diamine) in **DOCD2** has decreased its *λ*_max_ at 667.730 nm with transition energy of 1.857 eV and 1.552 *f*_*os*_. In this case. the major molecular orbitals contributions in this case are recorded as 84% for HOMO-1 to LUMO. The *λ*_max_ is further decreased in **DOCD3** upon introducing (4-(cyclopenta[b]indol-4(3H,4aH,8aH)-yl)-N-(4-(3,3a-dihydrocyclpenta[b]indol-4(4aH)-yl)phenyl)-N-phenylaniline) donor at 659.070 nm. Oscillation strength of 1.309 and 1.881 eV transition energy with 81% HOMO-1 to LUMO contributions. However, on introducing (N-(4-(4aH-carbazol-9(4bH,8aH,9aH)-yl)phenyl)-4-(8aH-carbazol-9(9aH)-yl)-N-phenylaniline) donor moiety in **DOCD4** has increased *λ*_max_ to 717.875 nm which is highest among the derivatives. This *λ*_max_ is red-shifted with the lowest transition energy of 1.727 eV and 1.779 *f*_*os*_, revealing 89% contributions of molecular orbitals from HOMO to LUMO. The *λ*_max_ has decreased to 706.38 and 700.79 nm in **DOCD5** and **DOCD6** due to the incorporation of (N-(4-(10H-phenothiazin-10-yl)phenyl)-4-(10H-phenothiazin-10-yl)-N-phenylaniline) and (N-(4-(10H-phenoxazin-10-yl)phenyl)-4-(10H-phenoxazin-10-yl-N-phenylaniline) donor moieties, respectively. The overall decreasing trend of TD-DFT computed *λ*_max_ values for the investigated compounds is found as **DOCD4** > **DOCD5** > **DOCD6** > **DOCR1** > **DOCD2** > **DOCD3**. From the above discussion, the highest efficiency of donor moiety in **DOCD4** results in the redshift and a decrease in the band gap. This shows that derivatives **DOCD4**–**DOCD6** have shown the highest charge transfer towards the acceptor from the donor via $\uppi$-linker. Compound **DOCD4** is remarkable and should be synthesized for use in optoelectronic devices.

### Study of natural bond orbitals (NBOs)

To interpret the nucleophilic and electrophilic hyper-conjugative interactions, other bonding interactions and mode of electronic transitions, NBO analysis is the most precise technique^52^. It is an important tool to investigate intra-molecular charge delocalization and its transference from occupied orbitals (D) to unfilled orbitals (A) in D–π–A^53^ framework. Table 3 shows combined data including all the possible electronic transitions, their types and the stabilization energies associated with these transitions for **DOCR1** and **DOCD2–DOCD6**.

**Table 3:** Natural bond orbital (NBO) investigation of compounds (**DOCR1** and **DOCD2**-**DOCD6**).

Compounds	Donor(i)	Type	Acceptor(j)	Type	E(2)^a^	E(J)E(i)^b^	F(i,j)^c^
**DOCR1**	C23–C32	π	C21–S30	π*	48.87	0.19	0.094
	C85–N86	π	C87–N88	π*	0.71	0.47	0.016
	C21–C32	σ	S17–C20	σ*	8.28	0.93	0.078
	C26–S29	σ	C26–C35	σ*	0.5	1.22	0.022
	C22	LP(1)	C38–C39	π*	70.65	0.18	0.118
	N88	LP(1)	C61–C87	σ*	12.65	1.04	0.103
**DOCD2**	C26–C27	π	C35–C36	π*	34.66	0.3	0.092
	C18–C20	π	C18–C20	π*	0.52	0.31	0.012
	C35–H37	σ	C26–S29	σ*	10.67	0.71	0.078
	C26–S29	σ	C26–C35	σ*	0.51	1.22	0.022
	N92	LP(1)	C93–C94	π*	13.35	0.3	0.058
	N113	LP(1)	C115–H 116	σ*	7.9	0.63	0.067
**DOCD3**	C26–C27	π	C35–C36	π*	34.48	0.3	0.092
	C68–N69	π	C70–N71	π*	0.72	0.47	0.017
	C35–H37	σ	C26–S29	σ*	10.63	0.71	0.077
	C22–S30	σ	C22–C23	σ*	0.5	1.25	0.022
	N114	LP(1)	C117–C118	π*	42.83	0.3	0.104
	O57	LP(2)	C55–C60	σ*	21.38	0.76	0.115
**DOCD4**	C26–C27	π	C35–C36	π*	34.33	0.3	0.092
	C68–N69	π	C70–N71	π*	0.72	0.47	0.016
	C35–H37	σ	C26–S29	σ*	10.62	0.71	0.077
	C96–C100	σ	N114–C125	σ*	0.51	1.16	0.022
	N113	LP(1)	C136–C137	π*	35.76	0.31	0.097
	O57	LP(2)	C36–C55	σ*	18.66	0.76	0.108
**DOCD5**	C26–C27	π	C35–C36	π*	34.28	0.3	0.092
	C103–C105	π	C85–C89	π*	0.54	0.3	0.012
	C35–H37	σ	C26–S29	σ*	10.61	0.71	0.077
	N113–C116	σ	C96–C100	σ*	0.51	1.36	0.024
	S29	LP(2)	C25–C31	π*	30.65	0.27	0.082
	N71	LP(1)	C56–C70	σ*	12.65	1.04	0.103
**DOCD6**	C26–C27	π	C35–C36	π*	34.26	0.3	0.092
	C25–C31	π	C25–C31	π*	1.5	0.29	0.019
	C35–H37	σ	C26–S29	σ*	10.62	0.71	0.077
	C22–S30	σ	C32–C34	σ*	0.51	1.1	0.021
	N114	LP(1)	C136–C137	π*	37.27	0.3	0.098
	O57	LP(2)	C55–C60	σ*	21.41	0.76	0.115

For evaluating the reactions involving delocalization, second-order perturbation approach is utilized. To measure the stabilization energy *E*^(2)^ in every single donor (i) to acceptor (j) transition, leading *i* $\to$ *j* delocalization the formula employed is:

4 $$E^{\left( 2 \right)a} = q_{{\text{i}}}^{b} \frac{{\left( {F_{{{\text{i}},{\text{j}}}}^{c} } \right)^{2} }}{{\varepsilon_{{\text{j}}} - \varepsilon_{{\text{i}}}^{d} }}$$

where *E*^(2)^ is the stabilization energy, *E*_*i*_ and *E*_*j*_ are diagonal element orbital energies, *q*_*i*_ is the donor-orbital occupancy and *F*_*i,j*_ is the Fock matrix element between the natural bonding orbitals of the entire structure^54^. Hyper-conjugation occurs due to the overlapping of the following orbitals: σ $\to$ σ*, π $\to$ π*, LP $\to$ σ* and LP $\to$ π. The π-conjugated systems like our designed D–π–A derivatives could be justified from their π → π* transitions credited as the most significant NLO materials. The other type of allowed transitions are feeble such as σ → σ* on account of weaker interactions between electron-rich donor and electron-deficient acceptor parts. The major values of these transitions are presented in Table 3 while, the detailed analysis is recorded in the supplementary information part (Tables S1–S6). In **DOCR1**, the highest value of stabilization energy in case of significant π $\to$ π* transitions is revealed at 48.87 kcal mol^−1^ exhibited by π (C23–C32) $\to$ π*(C21–S30). While, the slightest value is shown in π(C85–N86)$\to$ π*(C87–N88) is 0.71 kcal mol^−1^.

High *E*^(2)^ corresponds to robust interaction among D and A with enhanced conjugation in the feeble σ $\to$ σ* transitions noted for **DOCR1** are σ(C21–C32)$\to$ σ*(S17–C20) and σ(C26–S29)$\to$ σ*(C26–C35) with energies as 8.28 and 0.50 kcal mol^−1^, accordingly. The lone pair transitions involved in stabilizing the reference are: LP(1)(C22)$\to$ π*(C38–C39) and LP(1)(N88)$\to$ σ*(C61–C87) acquiring energies of 70.65 and 12.65 kcal mol^−1^, respectively. In **DOCD2,** the highest stability *i.e.* 34.66 kca mol^−1^ corresponds to π(C26–C27)$\to$ π*(C35–C36). While the lowest π(C18–C20) $\to$ π*(C18–C20) stabilization energy is 0.52 kcal mol^−1^. For σ $\to$ σ* transition, the highest stabilization energy is 10.67 kcal mol^−1^ obtained for σ(C35–H37) $\to$ σ*(C26–S29) while σ(C26–S29) $\to$ σ*(C26–C35) transitions corresponds to the lowest energy of 0.51 kcal mol^−1^. Other transitions *i.e.,* LP(1)(N92) $\to$ π*(C93–C94) and LP(1)(N113) $\to$ σ*(C115–H116) have energies of 13.35 and 7.9 kcal mol^−1^, respectively. In **DOCD3**, the maximum and minimum energies relative to π $\to$ π* are due to π(C26–C27) $\to$ π*(C35–C36) and π(C68–N69) $\to$ π*(C70–N71) at 34.48 and 0.72 kcal mol^−1^, respectively. While for σ $\to$ σ*, the maximum energy is 10.63 kcal mol^−1^ due to transitions among σ(C35–H37) $\to$ σ*(C26–S29). σ(C22–S30) $\to$ σ*(C22–C23) transition possess minimum stabilization energy of 0.5 kcal mol^−1^. The highest energy in lone pair transitions is due to LP(1)(N114) $\to$ π*(C117–C118) of 42.83 kcal mol^−1^. The lowest energy corresponds to transition involving LP(2)(O57) $\to$ σ*(C55–C60) of 21.38 kcal mol^−1^ stabilization energy. In **DOCD4**, π $\to$ π* involving transitions at π(C26–C27) $\to$ π*(C35–C36) with maximum energy of 34.33 kcal mol^−1^. While the minimum energy of 0.72 kcal mol^−1^ corresponds to π(C68–N69) $\to$ π*(C70–N71) transitions. Transitions due to σ(C35–H37) $\to$ σ*(C26–S29) has energy of 10.62 kcal mol^−1^, its minimum energy is due to σ(C96–C100) $\to$ σ*(N114–C125) at 0.51 kcal mol^−1^. The lone pair transitions have the highest energy due to LP(1)(N113) $\to$ π*(C136–C137) of 35.76 kcal mol^−1^. However, the lowest energy relates to transition involving LP(2)(O57) $\to$ σ*(C36–C55) of 18.66 kcal mol^−1^. In **DOCD5**, transitions relative to π $\to$ π* are due to π(C26–C27) $\to$ π*(C35–C36) and π(C103–C105) $\to \hspace{0.17em}$π*(C85–C89) at 34.28 and 0.54 kcal mol^−1^ are the maximum and minimum energies, respectively. Maximum energy σ(C35–H37) $\to$ σ*(C26–S29) transitions is at 10.61 kcal mol^−1^. While the minimum energy σ(N113–C116) $\to$ σ*(C96–C100) transition occurs at 0.51 kcal mol^−1^. The lone pair transitions have the highest energy transition from LP(2)(S29)  $\to$ π*(C25–C31) of 30.65 kcal mol^−1^. However, the lowest energy transition involve LP(1)(N71) $\to$ σ*(C56–C70) of 12.65 kcal mol^−1^. In last derivative **DOCD6**, the following important electronic transitions occur: π(C26–C27) $\to$ π*(C35–C36), π(C25–C31) $\to$ π*(C25–C31), σ(C35–H37) $\to$ σ*(C26–S29), σ(C22–S30) $\to$ σ*(C32–C34), LP(1)(N114) $\to$ π*(C136–C137) and LP(2)(O57) $\to$ σ*(C55–C60) with stabilization energy values as: 34.26, 1.50, 10.62, 0.51, 37.27 and 21.41 kcal mol^−1^, respectively. It is seen from the above mentioned analysis that the non-covalent interaction between filled and unfilled orbitals played a significant role in stabilizing the **DOCR1** and **DOCD2–DOCD6.**

Table 4 depicts the Mulliken charges associated with the donors, π-linkers and acceptor moieties of the studied compounds. It is seen from the calculated values that all the donors possess positive charges and the acceptors attained negative charges strongly supporting the intramolecular charge transfer within the donor–π–acceptor framework. Interestingly, π-spacers possess positive charges due to which they work as a charge facilitator. Consequently, the NBO study reveals that hyper-conjugation and effective intramolecular charge transference are important in molecular stability to implicate charge-shifting characteristics essential for NLO materials.

**Table 4:** NBO charges for **DOCR1** and **DOCD2–DOCD6**.

Compounds	Donor	π-spacer	Acceptor
DOCR1	–	0.506	− 0.574
DOCD2	0.119	0.162	− 0.282
DOCD3	0.098	0.180	− 0.278
DOCD4	0.077	0.197	− 0.274
DOCD5	0.071	0.202	− 0.273
DOCD6	0.071	0.201	− 0.272

### Global reactivity parameters (GRPs)

The *E*_*HOMO*_ and *E*_LUMO_ together with the band gap can be utilized to depict the reactivity and stability of compounds to predict chemical reactivity parameters^54,55^. These include electronegativity (*X*)^33^, ionization potential (*IP*), global softness (*σ*), electron affinity (*EA*), global hardness (*η*)^34^, electrophilicity index (*ω*)^35^ and chemical potential (*μ*). Ionization potential is the energy required to eliminate an electron from the highest occupied MO. While, the electron affinity is defined as the amount of energy liberated upon the addition of an electron to the lowest unoccupied MO^56^. The capability of an atom to attract the shared pair of electrons towards itself is its electronegativity^57^. Global reactivity parameters can be calculated using the Eqs. S1–S7^58,59^ which are given in supplementary file.

It has been noticed that the stability of the compound is directly influenced by the hardness (*η)* while, the softness (*σ*) is directly related to its reactivity. Molecular stability corresponds with the *µ* negative integer^60^. Compounds with higher global hardness values are least reactive and more stable. On the other hand, compounds having higher value of global softness are more reactive and are unstable. Among all the designed compounds, **DOCD2** exhibits smaller band gap (1.657 eV) with the highest value of softness (0.604 *E*_*h*_) and least hardness (0.829 *E*_*h*_) (Table 5). It is predicted that the **DOCD2** compound is the most reactive and shows remarkable NLO response. Global softness values for other compounds **DOCR1** and **DOCD3–DOCD6** are: 0.425, 0.560, 0.484, 0.473 and 0.471 *E*_*h*_, respectively. While, their global hardness is as follows: 1.176, 0.894, 1.033, 1.058 and 1.061 *E*_*h*_, respectively. The reactivity trend of the reference and all the derivatives in descending order is: **DOCD2** > **DOCD3** > **DOCD4** > **DOCD5** > **DOCD6** > **DOCR1**. The ionization potential of designed molecules ranges from (4.894–5.379 *E*_*h*_) and electron affinity values range from (3.237–3.258 *E*_*h*_). Among all the designed molecules, **DOCD2** exhibits lower band gap with high reactivity indicating polarization and good NLO response.

**Table 5:** The global reactivity descriptors of all the entitled compounds.

Compounds	*I*	*A*	*X*	$\eta$	$\mu$	$\omega$	$\sigma$
DOCR1	5.757	3.405	4.581	1.176	− 4.581	8.922	0.425
DOCD2	4.894	3.237	4.066	0.829	− 4.066	9.975	0.604
DOCD3	5.033	3.246	4.140	0.894	− 4.140	9.589	0.560
DOCD4	5.320	3.255	4.288	1.033	− 4.288	8.902	0.484
DOCD5	5.374	3.258	4.316	1.058	− 4.316	8.803	0.473
DOCD6	5.379	3.257	4.318	1.061	− 4.318	8.787	0.471

Units in *Hartree* (*E*_*h*_).

### Hole-electron interaction analysis

Hole-electron interaction analysis offers a deeper understanding of the nature of electron excitations in a molecule^61^. Multiwfn 3.8. was used to perform electron excitation analysis^62,63^. Figure S2 shows that in the reference molecule, a hole is produced at the C atom of the 5,5-dimethylcyclopenta-1,3-diene ring of the π-linker. At the same time, a significant electronic cloud can be observed over the thiophene ring (S atoms) of the π-bridge. The reason behind this could be the presence of the powerful electron-withdrawing ability of the Sulphur group. Noticeably, it is observed that a hole is induced in various atoms of the π-spacer, consecutively moving towards the acceptor region, demonstrating proficient charge transference from the π-linker towards the acceptor group under the effect of the electron donating group in all the designed compounds.

Further, Fig. S2 also reveals high-intensity holes at different atoms of the π-linker and charge is transferred at the acceptor region and studied maximum over the C atoms of the methylene group, which further linked with the strong electron-withdrawing cyano groups and resulted in an efficient ICT in all the derivatives (**DOCD2–DOCD6**). Overall, in investigated compounds (**DOCR1** and **DOCD2–DOCD4**), the electron intensity is detected to be maximum at the electronic band compared to the hole; therefore, they seem to be electron rich materials (Fig. S2). However, **DOCD5** and **DOCD6** are hole-type materials because the hole intensity ratio is higher at the hole band gap in these compounds.

### Transition density matrix (TDM) and binding energy (*E*_b_) analysis

**Figure 7:**
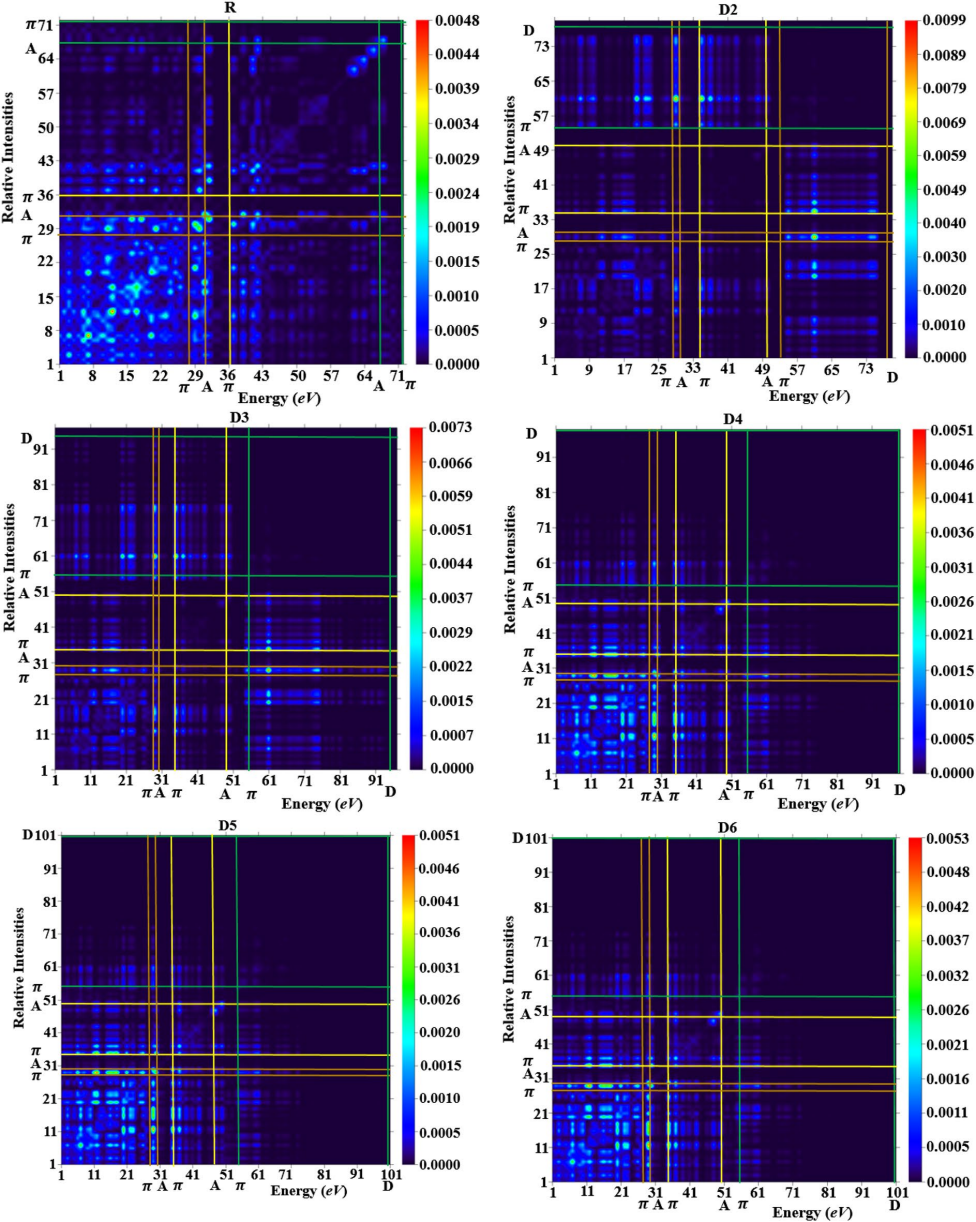
TDM graphs of compounds (**DOCR1** and **DOCD2**–**DOCD6)**. These heat maps were drawn with the help of Multiwfn 3.7 software (http://sobereva.com/multiwfn/). All out put files of designed compounds were accomplished by Gaussian 09 version D.01 (https://gaussian.com/g09citation/).

TDM is an essential tool for observing the charge transference in reference (**DOCR1**) and designed compounds (**DOCD2–DOCD6**)^51^. TDM aids in calculating the excitation of charge density, localization and delocalization of electron–hole pairs and the relation between electron-accepting and donating entities in the excited state^64–66^. In this work, the impact of the hydrogen (H) atom is neglected owing to its minute involvement in transitions. The TDM heat maps of every single designed entity manifest the nature of the electronic transition. The TDM outcomes of all the studied reference and derivatives are presented in Fig. 7.

To factor in the transfer of electronic charge, we distributed our studied compounds into three segments such as donor, π-spacer, and acceptor. TDM pictographs illustrate a reasonable proportion of diagonal electronic charge transference (CT) in all the designed chromophores. From comparative study of TDM heat maps of all the compounds (**DOCR1** and **DOCD2-DOCD6**) it is observed that they exhibit almost similar behavior. TDM pictographs in S_0_–S_1_ energy level (Fig. 7) confirm that electrons are significantly shifted from π-spacer to the acceptor counterparts which accelerate the transfer of electrons without any restriction. The results of TDM heat maps suggest schematic separation in the excited transition state that is significant for the production of NLO materials. The difference between electrical and optical band gap energies is called binding energy, which is a major tool to determine the optoelectronic characteristics of the designed compounds. Equation (5) is employed to estimate the binding energy of the reference and designed chromophores^67^.

5 $$E_{{\text{b}}} = E_{{{\text{L}}{ - }{\text{H}}}} - E_{{{\text{opt}}}}$$

In Eq. (5) *E*_b_ shows the binding energy, *E*_L−H_ indicates the band gap and *E*_opt_ depicts the first excitation energy^6,9^. The calculated outcomes of binding energy are displayed in Table 6.

**Table 6:** Calculated LUMO–HOMO energy gap (*E*_LUMO-HOMO_), first singlet excitation energy (*E*_opt_) and exciton binding energy (*E*_b_).

Compounds	*E*_L-H_	*E*_*op*t_	*E*_b_
DOCR1	2.325	1.814	0.511
DOCD2	1.657	1.857	− 0.20
DOCD3	1.787	1.881	− 0.094
DOCD4	2.065	1.727	0.338
DOCD5	2.116	1.755	0.361
DOCD6	2.122	1.769	0.353

Units in *eV.*

Table 6 shows that all the investigated compounds show smaller binding energies (0.361–0.20 eV) than the reference **DOCR1** (0.511 eV). These values could be due to the alteration in the configuration that establishes a strong push–pull alignment. Correspondingly, the exciton binding energy values of **DOCD2–DOCD6** are smaller than that of **DOCR1** with a comparable LUMO–HOMO energy gap sequence. This lower binding energy and smaller first excitation energy and *E*_gap_ values assist the large exciton dissociation and remarkably greater charge movement with improved optoelectronic characteristics^10^. The overall descending trend of binding energies of reference and designed chromophores is: **DOCR1 > DOCD5 > DOCD6 > DOCD4 > DOCD2 > DOCD3**. Binding energy relates to polarizability, and those with less binding energy are considered ideal photonic compounds with outstanding NLO responses^10^. Interestingly, the lowest binding energy (− 0.20 eV) of **DOCD2** owing to the high charge transport rate and ease of segregation into individual charges makes it an excellent NLO material.

### Nonlinear optical (NLO) properties

Improved nonlinear optical (NLO) properties in many substances are useful for emerging applications in the growing areas of harmonic generation, electro-optic modulation, frequency blending and in communications^13,68,69^. Therefore, sufficient comprehension of NLO characteristics is necessary to design such materials. Magnitude of optical response is determined by material’s electronic properties and influenced by polarizability (linear, *α*) and hyperpolarizability (nonlinear, *β* and $\upgamma$, etc.) and the dipole moment (*μ*_*tot*_)^52^, which is greatly influenced by the electronegativity of molecules. Computed data of dipole moment (*μ*_*tot*_) for the studied compounds (**DOCR1** and **DOCD2–DOCD6**) is available in Table S8 (calculated in Debye). The dipole moment tensor along the z-axis (*μ*_*z*_) shows the major contribution towards *μ*_*tot*_ values while, the values along the x and y-axis are small. The dipole moment values for these compounds are found in order **DOCD2** > **DOCD3** > **DOCD4** > **DOCD5** > **DOCD6** > **DOCR1**. The derivative **DOCD2** shows the highest value and is considered as the most polarized molecule.

Likewise, the linear polarizability <$\alpha$> effectively describes the electronic properties of compounds along-with their polarity. The <$\alpha$> values along with their major contributing factors are enlisted in Tables S7–S10 while, the major values are presented in Table 7 of the manuscript (all parameters in esu unit). The average polarizability tensor along x-axis $({\alpha }_{x})$ values are dominant among all other tensor components, indicating that <$\alpha$> lie along this direction. The measurements for average polarizability confirms that average polarizability is dominant in the derivative **DOCD5** (3.114 × 10^–22^ esu) with *α*_xx_ = 5.438 × 10^–22^ esu, *α*_yy_ = 2.552 × 10^–22^ esu and *α*_zz_ = 1.352 × 10^–22^ esu as x, y and z-axis parameters, respectively. It has been noted that *α*_xx_ is the major contributing factor in the overall value of <$\alpha$>. It is known from literature that the energy gap between LUMO and HOMO influences the polarizability of a molecule. The molecules with small energy gap values possess significant linear polarizability.

**Table 7:** The computed polarizabilities < $\alpha$>, first (*β*_*tot*_) and second hyperpolarizabilities ($\upgamma$_*tot*_*)* of the studied compounds (in esu) **DOCR1** and **DOCD2**–**DOCD6**.

Compounds	<$\alpha$> × 10^–22^	*β*_*tot*_ × 10^–27^	$\upgamma$_*tot*_ × 10^–31^
DOCR1	2.646	0.493	0.535
DOCD2	2.767	7.184	1.676
DOCD3	3.106	5.691	1.144
DOCD4	3.097	4.345	0.712
DOCD5	3.114	0.003	0.631
DOCD6	3.015	3.736	0.588

Utilizing transfer of charge (CT) among electron-donating and extracting motifs so to reduce the band gap by designing new D–π–A framework that increases the first hyperpolarizability (*β*_*tot*_)^70^. The NLO response of designed compounds is highlighted by determining their first hyperpolarizability (*β*_*tot*_) values. The computed data for the first hyperpolarizability values of compounds (**DOCR1** and **DOCD2–DOCD6**) along with their tensor components is tabulated in Table S9. Among all the designed compounds, **DOCD2** shows dominant *β*_*tot*_ value (7.184 × 10^–27^ esu) which could be attributed to the well-established electronic communication within its push–pull architecture. The major contributing tensor for compounds **DOCR1** and **DOCD2** is *β*_yyy_ *i.e.* along y-axis displaying magnitude of 0.028 × 10^–27^ and 0.020 × 10^–27^ esu, respectively. For **DOCD3** and **DOCD4**, the dominating tensor is located at x-plane (*β*_xxx_) with values of 5.621 × 10^–27^ and 4.311 × 10^–27^ esu, respectively. *β*_xzz_ contributes the most to the *β*_*tot*_ value in **DOCD5** (*β*_xzz_ = 0.004 × 10^−27^ esu) and **DOCD6** (*β*_xzz_ = 0.007 × 10^–27^ esu) chromophores. Generally, first hyperpolarizability is directly related with linear polarizability values and inversely related with energy gap values^71^. In the present case, *β*_*tot*_ values are in good agreement with the band gap trends, highest *β*_*tot*_ value (7.184 × 10^–27^ esu) is exhibited by the compound with smallest band gap (1.657 eV).

The second hyperpolarizability $\upgamma$_*tot*_ values for the investigated compounds were also calculated using M06 method with 6-311G (d,p) basis set are displayed in Table S10. According to the data obtained, the major contribution in $\upgamma$_*tot*_ values is done by the second hyperpolarizability tensor along x-axis ($\upgamma$_x_) in all the entitled compounds. Compound **DOCD2** (1.667 × 10^–31^ esu) is found with highest $\upgamma$_*tot*_ value with dominant tensor $\upgamma$_x_ = 1.667 esu while the tensor along z-axis ($\upgamma$_***z***_) displayed least contribution towards $\upgamma$_*tot*_ in the same compound (0.0001 × 10^–31^ esu)^72^. A comparative analysis is made among the **DOCR1** and **DOCD2-DOCD6** and urea molecule which is used as a standard compound in order to examine the NLO response of photonic materials^73^. By comparing the NLO findings of **DOCR1** and **DOCD2-DOCD6** with standard, we came to know that *β*_*tot*_ value of **DOCD2** compound is found as 1.931 × 10^–56^ times greater than that of urea which is equal to 0.372 × 10^–30^ esu^74^. The computed statistics obtained from comparative analysis with urea highlighted that designed compounds possess appreciable NLO characteristics suggesting that they may prove to be suitable NLO materials. On attaining the maximum values of $\mu$_*tot*_, *β*_*tot*_ and $\upgamma$_*tot*_, the compound **DOCD2** is nominated as the potential NLO material in emerging NLO-related technology.

## Conclusion

Herein, some unique non-fullerene ring compounds (**DOCD2-DOCD6**) have been designed with D–π–A architecture using the **DOCR1**. The central core acts as a π-spacer along with a terminal acceptor at one end, and the other end is modified with various donor moieties. Surprisingly, all the derivatives were found to have less HOMO–LUMO band gap than the reference (**DOCR1)** with the following increasing order: **DOCD2** ˂ **DOCD3** ˂** DOCD4 **˂** DOCD5 **˂** DOCD6 **˂** DOCR1**. Their UV–Vis spectra also reported stronger absorption wavelengths (700.792–717.875 nm) with correspondingly lower transition energies. The binding energy (*E*_b_) values indicated that donor moieties play a key role in decreasing these values. The compounds showed lower *E*_b_ values (− 0.20 to 0.361 eV) than the reference **DOCR1** (0.511 eV) which infer that less Columbic forces with enhanced coherence electron transmission were noticed in bridge and acceptor motifs. The values of <$\alpha$>, *β*_*total*_ and *γ*_*total*_ are remarkable for designed derivatives compared to **DOCR1**. Interestingly, promising results are obtained in the case of **DOCD2** (<$\alpha$>  = 2.767 × 10^–22^, *β*_*total*_ = 7.184 × 10^–27^ and *γ*_*total*_ = 1.676 × 10^–31^ esu). To conclude all our DFT computations, the effective strategies utilized in the designing lead to better entrants for NLO, which could have prospective applications in advancing the technology.

## Supplementary Information

Supplementary Information.

## Data Availability

All data generated and analyzed during this study are included in this published article and its supplementary information files.
