# Supplementary material for: Effect of different end-capped donor moieties on non-fullerenes based non-covalently fused-ring derivatives for achieving high-performance NLO properties
Source: Sci Rep. 2023 Jan 25;13:1395. doi: 10.1038/s41598-023-28118-w (PMC9876985; doi:10.1038/s41598-023-28118-w)
Supplement: Supplementary file 1 — Supplementary Information. [file 41598_2023_28118_MOESM1_ESM.docx]

**Supplementary Information**

**Effect of Different End-Capped Donor Moieties on Non-fullerenes and Non-Covalently Fused-Ring Derivatives for Achieving High-Performance NLO Devices**

Muhammad Khalid*,^1,2^ Iqra Shafiq,^1,2^ Umm-e-Hani^1,2^ Khalid Mahmood,^3^ Riaz Hussain.^4^ Muhammad Fayyaz ur Rehman,^5^ Mohammed A. Assiri,^6,7^ Muhammad Imran,^6,7^ Muhammad Safwan Akram*^8,9^

^1^Institute of Chemistry, Khwaja Fareed University of Engineering & Information Technology, Rahim Yar Khan, 64200, Pakistan

^2^Centre for Theoretical and Computational Research, Khwaja Fareed University of Engineering & Information Technology, Rahim Yar Khan, 64200, Pakistan

^3^Institute of Chemical Sciences, Bahauddin Zakariya University, Multan 60800, Pakistan

^4^Department of Chemistry, Division of Science and Technology, University of Education Lahore, Pakistan

^5^Department of Chemistry, University of Sargodha, Sargodha, Pakistan

^6^Department of Chemistry, Faculty of Science, King Khalid University, P.O. Box 9004, Abha 61413, Saudi Arabia.

^7^Research center for Advanced Materials Science (RCAMS), king Khalid University, Abha 61514, P. O. Box 9004, Saudi Arabia.

^8^National Horizons Centre, Teesside University, Darlington, DL11HG, United Kingdom.

^9^School of Health & Life Sciences, Teesside University, Middlesbrough, TS1 3BX, United Kingdom

*Corresponding author E-mail addresses:

Dr. Muhammad Khalid (muhammad.khalid@kfueit.edu.pk; [Khalid@iq.usp.br](mailto:Khalid@iq.usp.br))

Dr. Muhammad Safwan Akram (Safwan.akram@tees.ac.uk)

**Table S1:** Second-order perturbation theory analysis of fock matrix in **DOCR1.**

| **Donor(i)** | **Type** | **Acceptor(j)** | **Type** | **E(2)^a^** | **E(J)E(i)^b^** | **F(i,j)^c^** |
| --- | --- | --- | --- | --- | --- | --- |
| C23-C32 | π | C21-S30 | π* | 48.87 | 0.19 | 0.094 |
| C27-C31 | π | C26-S29 | π* | 38.94 | 0.17 | 0.086 |
| C18-C20 | π | C21-S30 | π* | 34.76 | 0.2 | 0.084 |
| C38-C39 | π | C59-C62 | π* | 27.64 | 0.3 | 0.081 |
| C38-C39 | π | C60-O64 | π* | 24.7 | 0.31 | 0.079 |
| C1-C6 | π | C2-C3 | π* | 22.69 | 0.3 | 0.075 |
| C76-C77 | π | C74-C75 | π* | 21.37 | 0.29 | 0.071 |
| C1-C6 | π | C4-C5 | π* | 18.8 | 0.31 | 0.07 |
| C18-C20 | π | C15-C16 | π* | 17.63 | 0.3 | 0.066 |
| C9-C10 | π | C1-C6 | π* | 15.48 | 0.31 | 0.065 |
| C26-S29 | π | C35-C36 | π* | 8.97 | 0.34 | 0.051 |
| C21-S30 | π | C23-C32 | π* | 8.7 | 0.36 | 0.053 |
| C57-C61 | π | C35-C36 | π* | 7.83 | 0.33 | 0.047 |
| C60-O64 | π | C76-C77 | π* | 4.13 | 0.42 | 0.042 |
| C87-N88 | π | C85-N86 | π* | 0.74 | 0.47 | 0.017 |
| C85-N86 | π | C87-N88 | π* | 0.71 | 0.47 | 0.016 |
| C21-C32 | σ | S17-C20 | σ* | 8.28 | 0.93 | 0.078 |
| C35-H37 | σ | C36-C58 | σ* | 6.81 | 0.99 | 0.074 |
| C66-H71 | σ | C67-C68 | σ* | 5.31 | 1.11 | 0.068 |
| C26-C27 | σ | C27-C31 | σ* | 4.59 | 1.31 | 0.069 |
| C23-C32 | σ | C21-C32 | σ* | 4.26 | 1.27 | 0.066 |
| C16-C18 | σ | C18-C20 | σ* | 4.16 | 1.27 | 0.065 |
| C21-S30 | σ | C22-C38 | σ* | 3.71 | 1.26 | 0.061 |
| C58-C67 | σ | C35-C36 | σ* | 3.36 | 1.27 | 0.058 |
| C57-C68 | σ | C67-C68 | σ* | 3.1 | 1.24 | 0.055 |
| C62-C89 | σ | C62-C91 | σ* | 2.59 | 1.26 | 0.051 |
| C36-C58 | σ | C36-C57 | σ* | 2.24 | 1.16 | 0.046 |
| C39-C60 | σ | C60-O64 | σ* | 2.06 | 1.26 | 0.046 |
| C16-C18 | σ | C16-H19 | σ* | 1.92 | 1.11 | 0.041 |
| C25-S29 | σ | C14-C25 | σ* | 1.44 | 1.23 | 0.038 |
| C39-C60 | σ | C60-C77 | σ* | 1.21 | 1.13 | 0.033 |
| S11-C14 | σ | C25-C31 | σ* | 1.07 | 1.24 | 0.033 |
| C33-C41 | σ | C12-C14 | σ* | 1.01 | 1.18 | 0.031 |
| C26-C35 | σ | C35-H37 | σ* | 0.98 | 1.14 | 0.03 |
| C5-H8 | σ | C4-H7 | σ* | 0.91 | 0.94 | 0.026 |
| C1-C2 | σ | O93-C95 | σ* | 0.83 | 1.02 | 0.026 |
| C60-O64 | σ | C39-C59 | σ* | 0.73 | 1.6 | 0.031 |
| C58-O63 | σ | C67-C68 | σ* | 0.66 | 1.67 | 0.03 |
| C95-H96 | σ | C1-O93 | σ* | 0.64 | 0.89 | 0.022 |
| C34-C53 | σ | C53-H55 | σ* | 0.58 | 1.01 | 0.022 |
| C26-S29 | σ | C26-C35 | σ* | 0.5 | 1.22 | 0.022 |
| C22 | LP(1) | C38-C39 | π* | 70.65 | 0.18 | 0.118 |
| S17 | LP(2) | C15-C16 | π* | 25.04 | 0.27 | 0.074 |
| S17 | LP(2) | C21-S30 | π* | 0.91 | 0.17 | 0.012 |
| N88 | LP(1) | C61-C87 | σ* | 12.65 | 1.04 | 0.103 |
| O94 | LP(2) | C1-C2 | σ* | 4.2 | 0.93 | 0.056 |
| F84 | LP(1) | C73-C74 | σ* | 0.95 | 1.59 | 0.035 |

**Table S2:** Second order Perturbation theory analysis of Fock matrix in **DOCD2.**

| **Donor(i)** | **Type** | **Acceptor(j)** | **Type** | **E(2)^a^** | **E(J)E(i)^b^** | **F(i,j)^c^** |
| --- | --- | --- | --- | --- | --- | --- |
| C26-C27 | π | C35-C36 | π* | 34.66 | 0.3 | 0.092 |
| C35-C36 | π | C54-C56 | π* | 28.48 | 0.29 | 0.082 |
| C96-C 100 | π | C93-C94 | π* | 27.2 | 0.29 | 0.08 |
| C35-C36 | π | C55-O57 | π* | 25.13 | 0.3 | 0.08 |
| C61-C62 | π | C58-C63 | π* | 24.6 | 0.28 | 0.074 |
| C82-C83 | π | C84-C87 | π* | 22.68 | 0.3 | 0.074 |
| C1-C6 | π | C2-C3 | π* | 21.32 | 0.3 | 0.073 |
| C15-C16 | π | C2-C3 | π* | 17 | 0.31 | 0.068 |
| C 9-C10 | π | C1-C6 | π* | 14.82 | 0.31 | 0.064 |
| C35-C36 | π | C26-C27 | π* | 11.51 | 0.29 | 0.053 |
| C54-C56 | π | C61-C62 | π* | 9.26 | 0.32 | 0.051 |
| C54-C56 | π | C35-C36 | π* | 7.78 | 0.33 | 0.047 |
| C55-O57 | π | C59-C60 | π* | 4.16 | 0.43 | 0.041 |
| C55-O57 | π | C35-C36 | π* | 3.53 | 0.43 | 0.038 |
| C25-C31 | π | C25-C31 | π* | 1.52 | 0.29 | 0.019 |
| C68-N69 | π | C70-N71 | π* | 0.72 | 0.47 | 0.017 |
| C18-C20 | π | C18-C20 | π* | 0.52 | 0.31 | 0.012 |
| C35-H37 | σ | C26-S29 | σ* | 10.67 | 0.71 | 0.078 |
| C56-C70 | σ | C70-N71 | σ* | 8.17 | 1.61 | 0.103 |
| C68-N69 | σ | C56-C68 | σ* | 7.96 | 1.57 | 0.101 |
| C35-H37 | σ | C36-C55 | σ* | 6.73 | 0.99 | 0.073 |
| C35-C36 | σ | C26-C35 | σ* | 5.86 | 1.31 | 0.078 |
| C9-C10 | σ | C10-C12 | σ* | 5.13 | 1.31 | 0.073 |
| C93-C94 | σ | C93-C95 | σ* | 4.94 | 1.3 | 0.071 |
| C14-C25 | σ | C12-C14 | σ* | 4.75 | 1.29 | 0.07 |
| C26-C27 | σ | C26-C35 | σ* | 4.58 | 1.28 | 0.068 |
| C16-C18 | σ | C15-C16 | σ* | 4.49 | 1.28 | 0.068 |
| C21-C32 | σ | C23-C32 | σ* | 4.28 | 1.27 | 0.066 |
| C105-C108 | σ | C108-C110 | σ* | 4.19 | 1.28 | 0.065 |
| C62-C63 | σ | C58-C63 | σ* | 3.97 | 1.27 | 0.064 |
| C54-C61 | σ | C56-C68 | σ* | 3.85 | 1.18 | 0.06 |
| S11-C14 | σ | C6-C 9 | σ* | 3.53 | 1.2 | 0.058 |
| C55-C60 | σ | C60-C61 | σ* | 2.9 | 1.23 | 0.053 |
| C103-C104 | σ | C105-H109 | σ* | 2.46 | 1.13 | 0.047 |
| C100-N 113 | σ | C96-C100 | σ* | 2.3 | 1.37 | 0.05 |
| C36-C54 | σ | C54-C61 | σ* | 2.01 | 1.15 | 0.043 |
| C9-C10 | σ | C10-H13 | σ* | 1.83 | 1.13 | 0.041 |
| C63-F66 | σ | C58-C59 | σ* | 1.17 | 1.65 | 0.039 |
| C100-N113 | σ | C119-H122 | σ* | 0.54 | 1.18 | 0.023 |
| C110-N114 | σ | C123-H126 | σ* | 0.53 | 1.18 | 0.022 |
| C16-C18 | σ | C32-C34 | σ* | 0.52 | 1.14 | 0.022 |
| C26-S29 | σ | C26-C35 | σ* | 0.51 | 1.22 | 0.022 |
| N92 | LP(1) | C93-C94 | π* | 13.35 | 0.3 | 0.058 |
| N92 | LP(1) | C103-C104 | π* | 12.6 | 0.3 | 0.056 |
| O73 | LP(1) | C2-C3 | π* | 4.32 | 0.59 | 0.05 |
| N113 | LP(1) | C115-H116 | σ* | 7.9 | 0.63 | 0.067 |
| O72 | LP(2) | C1-C6 | σ* | 6.67 | 0.93 | 0.071 |
| O72 | LP(2) | C74-H75 | σ* | 5.83 | 0.72 | 0.059 |
| N113 | LP(1) | C119-H120 | σ* | 4.82 | 0.63 | 0.053 |

**Table S3:** Second order Perturbation theory analysis of Fock matrix in **DOCD3.**

| **Donor(i)** | **Type** | **Acceptor(j)** | **Type** | **E(2)^a^** | **E(J)E(i)^b^** | **F(i,j)^c^** |
| --- | --- | --- | --- | --- | --- | --- |
| C26-C27 | π | C35-C36 | π* | 34.48 | 0.3 | 0.092 |
| C35-C36 | π | C54-C56 | π* | 28.42 | 0.29 | 0.082 |
| C117-C118 | π | C119-C120 | π* | 25.57 | 0.3 | 0.079 |
| C61-C62 | π | C58-C63 | π* | 24.62 | 0.28 | 0.074 |
| C98-C100 | π | C93-C95 | π* | 23.51 | 0.3 | 0.075 |
| C140-C141 | π | C142-C143 | π* | 22.36 | 0.31 | 0.075 |
| C104-C106 | π | C108-C110 | π* | 21.44 | 0.3 | 0.073 |
| C104-C106 | π | C103-C105 | π* | 19.96 | 0.3 | 0.071 |
| C4-C5 | π | C1-C6 | π* | 18.68 | 0.3 | 0.069 |
| C59-C60 | π | C55-O57 | π* | 17.74 | 0.31 | 0.068 |
| C82-C83 | π | C22-C23 | π* | 15.3 | 0.29 | 0.059 |
| C25-C31 | π | C12-C14 | π* | 14.58 | 0.3 | 0.06 |
| C54-C56 | π | C61-C62 | π* | 9.26 | 0.32 | 0.051 |
| C54-C56 | π | C35-C36 | π* | 7.78 | 0.33 | 0.047 |
| C70-N71 | π | C68-N69 | π* | 0.74 | 0.47 | 0.017 |
| C68-N69 | π | C70-N71 | π* | 0.72 | 0.47 | 0.017 |
| C35-H37 | σ | C26-S29 | σ* | 10.63 | 0.71 | 0.077 |
| C18-C20 | σ | C21-S30 | σ* | 8.33 | 0.92 | 0.078 |
| C68-N69 | σ | C56-C68 | σ* | 7.95 | 1.57 | 0.101 |
| C22-C23 | σ | C32-C34 | σ* | 6.63 | 1.17 | 0.079 |
| C115-C120 | σ | C116-C129 | σ* | 5.28 | 1.15 | 0.07 |
| C108-H112 | σ | C106-C110 | σ* | 4.46 | 1.1 | 0.063 |
| C104-C106 | σ | C103-C104 | σ* | 4.37 | 1.3 | 0.067 |
| C3-C15 | σ | C15-C16 | σ* | 4.22 | 1.28 | 0.066 |
| C85-C89 | σ | C83-C85 | σ* | 4.1 | 1.31 | 0.066 |
| C15-S17 | σ | C16-H19 | σ* | 4.09 | 1.06 | 0.059 |
| C143-C150 | σ | C142-C143 | σ* | 4.03 | 1.24 | 0.063 |
| C148-C149 | σ | C150-H156 | σ* | 1.2 | 1.02 | 0.031 |
| C148-H153 | σ | N113-C149 | σ* | 1.04 | 0.84 | 0.026 |
| C34-C46 | σ | C18-C20 | σ* | 0.94 | 1.19 | 0.03 |
| C62-H65 | σ | C62-C63 | σ* | 0.91 | 1.1 | 0.028 |
| C149-H155 | σ | C143-C150 | σ* | 0.89 | 0.97 | 0.026 |
| C151-C152 | σ | C150-C151 | σ* | 0.86 | 0.99 | 0.026 |
| C84-H88 | σ | C87-H91 | σ* | 0.82 | 0.94 | 0.025 |
| C5-H8 | σ | C5-C6 | σ* | 0.76 | 1.08 | 0.026 |
| C150-C151 | σ | C151-H157 | σ* | 0.74 | 1.01 | 0.024 |
| C21-C32 | σ | C21-S30 | σ* | 0.72 | 0.92 | 0.023 |
| C148-H154 | σ | C152-H159 | σ* | 0.54 | 0.9 | 0.02 |
| C126-C127 | σ | C127-H134 | σ* | 0.52 | 1 | 0.02 |
| C22-S30 | σ | C22-C23 | σ* | 0.5 | 1.25 | 0.022 |
| N114 | LP(1) | C117-C118 | π* | 42.83 | 0.3 | 0.104 |
| S29 | LP(2) | C25-C31 | π* | 30.65 | 0.27 | 0.082 |
| S11 | LP(2) | C9-C10 | π* | 25.33 | 0.27 | 0.074 |
| S29 | LP(2) | C26-C27 | π* | 21.23 | 0.26 | 0.067 |
| O57 | LP(2) | C55-C60 | σ* | 21.38 | 0.76 | 0.115 |
| N69 | LP(1) | C56-C68 | σ* | 12.61 | 1.04 | 0.102 |
| O72 | LP(2) | C74-H77 | σ* | 4.48 | 0.73 | 0.052 |
| O73 | LP(1) | C1-C2 | σ* | 3.66 | 1.13 | 0.058 |

**Table S4:** Second order Perturbation theory analysis of Fock matrix in **DOCD4.**

| **Donor(i)** | **Type** | **Acceptor(j)** | **Type** | **E(2)^a^** | **E(J)E(i)^b^** | **F(i,j)^c^** |
| --- | --- | --- | --- | --- | --- | --- |
| C26-C27 | π | C35-C36 | π* | 34.33 | 0.3 | 0.092 |
| C35-C36 | π | C54-C56 | π* | 28.28 | 0.29 | 0.082 |
| C35-C36 | π | C55-O57 | π* | 25.01 | 0.31 | 0.08 |
| C2-C3 | π | C1-C6 | π* | 23.98 | 0.3 | 0.077 |
| C118-C119 | π | C115-C120 | π* | 22.87 | 0.3 | 0.074 |
| C59-C60 | π | C61-C62 | π* | 22.02 | 0.3 | 0.073 |
| C9-C10 | π | C12-C14 | π* | 21.21 | 0.31 | 0.076 |
| C148-C149 | π | C146-C147 | π* | 19.64 | 0.29 | 0.069 |
| C2-C3 | π | C4-C5 | π* | 18.41 | 0.31 | 0.069 |
| C25-C31 | π | C12-C14 | π* | 14.55 | 0.3 | 0.06 |
| C35-C36 | π | C26-C27 | π* | 11.45 | 0.29 | 0.053 |
| C54-C56 | π | C61-C62 | π* | 9.27 | 0.32 | 0.051 |
| C55-O57 | π | C59-C60 | π* | 4.15 | 0.43 | 0.041 |
| C70-N71 | π | C68-N69 | π* | 0.74 | 0.47 | 0.017 |
| C68-N69 | π | C70-N71 | π* | 0.72 | 0.47 | 0.016 |
| C35-H37 | σ | C26-S29 | σ* | 10.62 | 0.71 | 0.077 |
| C12-C14 | σ | C25-S29 | σ* | 8.1 | 0.94 | 0.078 |
| C68-N69 | σ | C56-C68 | σ* | 7.96 | 1.57 | 0.101 |
| C115-C116 | σ | C116-C117 | σ* | 5.65 | 1.28 | 0.076 |
| C36-C54 | σ | C35-C36 | σ* | 5.49 | 1.29 | 0.075 |
| C82-C83 | σ | C22-C23 | σ* | 2.56 | 1.29 | 0.051 |
| N114-C116 | σ | N114-C125 | σ* | 2.5 | 1.23 | 0.05 |
| C33-C38 | σ | C31-C33 | σ* | 2.03 | 1.05 | 0.041 |
| N92-C93 | σ | C103-C105 | σ* | 1.98 | 1.36 | 0.046 |
| C3-C15 | σ | C4-C5 | σ* | 1.86 | 1.31 | 0.044 |
| C22-C23 | σ | C23-H24 | σ* | 1.81 | 1.13 | 0.041 |
| C18-C34 | σ | C34-C50 | σ* | 1.7 | 1.01 | 0.037 |
| C2-C3 | σ | O73-C78 | σ* | 1.65 | 1.01 | 0.037 |
| S17-C20 | σ | C20-C21 | σ* | 1.35 | 1.21 | 0.036 |
| C21-S30 | σ | C20-C21 | σ* | 1.21 | 1.22 | 0.034 |
| C5-C6 | σ | C5-H8 | σ* | 1.09 | 1.12 | 0.031 |
| C33-C42 | σ | C12-C14 | σ* | 0.99 | 1.18 | 0.031 |
| C115-H121 | σ | C115-C116 | σ* | 0.91 | 1.11 | 0.028 |
| C12-C14 | σ | C33-C38 | σ* | 0.86 | 1.1 | 0.028 |
| C136-C149 | σ | N113-C137 | σ* | 0.82 | 1.1 | 0.027 |
| C120-H124 | σ | C115-C120 | σ* | 0.72 | 1.11 | 0.025 |
| C55-O57 | σ | C60-C61 | σ* | 0.66 | 1.67 | 0.03 |
| C10-C12 | σ | C31-C33 | σ* | 0.54 | 1.14 | 0.022 |
| C16-C18 | σ | C32-C34 | σ* | 0.53 | 1.14 | 0.022 |
| C96-C100 | σ | N114-C125 | σ* | 0.51 | 1.16 | 0.022 |
| N113 | LP(1) | C136-C137 | π* | 35.76 | 0.31 | 0.097 |
| N92 | LP(1) | C103-C104 | π* | 22.02 | 0.29 | 0.074 |
| O72 | LP(1) | C1-C6 | π* | 4.07 | 0.59 | 0.048 |
| O57 | LP(2) | C36-C55 | σ* | 18.66 | 0.76 | 0.108 |
| O73 | LP(2) | C1-C2 | σ* | 4.68 | 0.92 | 0.059 |
| O73 | LP(1) | C78-H81 | σ* | 2.82 | 0.94 | 0.047 |

**Table S5:** Second order Perturbation theory analysis of Fock matrix in **DOCD5.**

| **Donor(i)** | **Type** | **Acceptor(j)** | **Type** | **E(2)^a^** | **E(J)E(i)^b^** | **F(i,j)^c^** |
| --- | --- | --- | --- | --- | --- | --- |
| C26-C27 | π | C35-C36 | π* | 34.28 | 0.3 | 0.092 |
| C35-C36 | π | C54-C56 | π* | 28.26 | 0.29 | 0.082 |
| C25-C31 | π | C26-C27 | π* | 27.32 | 0.28 | 0.08 |
| C115-C120 | π | C116-C117 | π* | 24.89 | 0.28 | 0.077 |
| C128-C129 | π | C124-C125 | π* | 21.94 | 0.31 | 0.075 |
| C1-C6 | π | C9-C10 | π* | 17.13 | 0.29 | 0.063 |
| C35-C36 | π | C26-C27 | π* | 11.44 | 0.29 | 0.053 |
| C54-C56 | π | C61-C62 | π* | 9.27 | 0.32 | 0.051 |
| C55-O57 | π | C59-C60 | π* | 4.15 | 0.43 | 0.041 |
| C55-O57 | π | C35-C36 | π* | 3.54 | 0.43 | 0.038 |
| C35-C36 | π | C35-C36 | π* | 2.97 | 0.31 | 0.027 |
| C68-N69 | π | C70-N71 | π* | 0.72 | 0.47 | 0.016 |
| C103-C105 | π | C85-C89 | π* | 0.54 | 0.3 | 0.012 |
| C35-H37 | σ | C26-S29 | σ* | 10.61 | 0.71 | 0.077 |
| C21-C32 | σ | S17-C20 | σ* | 8.06 | 0.93 | 0.078 |
| C26-C27 | σ | C31-C33 | σ* | 7.3 | 1.15 | 0.082 |
| C54-C56 | σ | C56-C70 | σ* | 6.09 | 1.27 | 0.079 |
| C1-C2 | σ | C2-C3 | σ* | 5.55 | 1.3 | 0.076 |
| C105-H109 | σ | C103-C104 | σ* | 4.81 | 1.1 | 0.065 |
| C5-C6 | σ | C4-C5 | σ* | 3.86 | 1.31 | 0.064 |
| C22-S30 | σ | C82-C84 | σ* | 2.97 | 1.23 | 0.054 |
| C25-C31 | σ | C31-C33 | σ* | 2.76 | 1.14 | 0.05 |
| C2-C3 | σ | C15-C16 | σ* | 2.21 | 1.3 | 0.048 |
| C98-C100 | σ | N113-C116 | σ* | 1.15 | 1.13 | 0.032 |
| C54-C56 | σ | C60-C61 | σ* | 1.04 | 1.33 | 0.033 |
| S17-C20 | σ | C21-C32 | σ* | 0.98 | 1.27 | 0.032 |
| C98-H102 | σ | C95-C98 | σ* | 0.93 | 1.11 | 0.029 |
| C25-C31 | σ | C33-C42 | σ* | 0.85 | 1.09 | 0.027 |
| C98-H102 | σ | C95-H99 | σ* | 0.8 | 0.93 | 0.024 |
| C120-H123 | σ | C119-C120 | σ* | 0.74 | 1.12 | 0.026 |
| C21-C32 | σ | C21-S30 | σ* | 0.72 | 0.92 | 0.023 |
| C78-H80 | σ | C2-O73 | σ* | 0.65 | 0.89 | 0.022 |
| C34-C46 | σ | C46-H47 | σ* | 0.6 | 1.01 | 0.022 |
| C33-C38 | σ | C38-H39 | σ* | 0.58 | 1.01 | 0.022 |
| N114-C147 | σ | C106-C110 | σ* | 0.53 | 1.36 | 0.024 |
| C22-S30 | σ | C22-C23 | σ* | 0.52 | 1.25 | 0.023 |
| N113-C116 | σ | C96-C100 | σ* | 0.51 | 1.36 | 0.024 |
| S29 | LP(2) | C25-C31 | π* | 30.65 | 0.27 | 0.082 |
| S29 | LP(2) | C26-C27 | π* | 21.28 | 0.26 | 0.067 |
| S153 | LP(2) | C134-C135 | π* | 12.91 | 0.29 | 0.058 |
| N71 | LP(1) | C56-C70 | σ* | 12.65 | 1.04 | 0.103 |
| N114 | LP(1) | C147-C148 | σ* | 0.7 | 0.84 | 0.023 |
| O72 | LP(2) | S11-C14 | σ* | 0.52 | 0.59 | 0.016 |

**Table S6:** Second order Perturbation theory analysis of Fock matrix in **DOCD6.**

| **Donor(i)** | **Type** | **Acceptor(j)** | **Type** | **E(2)^a^** | **E(J)E(i)^b^** | **F(i,j)^c^** |
| --- | --- | --- | --- | --- | --- | --- |
| C26-C27 | π | C35-C36 | π* | 34.26 | 0.3 | 0.092 |
| C25-C31 | π | C26-C27 | π* | 27.3 | 0.28 | 0.08 |
| C103-C105 | π | C108-C110 | π* | 24.66 | 0.3 | 0.077 |
| C85-C89 | π | C82-C83 | π* | 22.66 | 0.3 | 0.075 |
| C134-C135 | π | C136-C137 | π* | 21.42 | 0.3 | 0.074 |
| C4-C5 | π | C1-C6 | π* | 19.05 | 0.3 | 0.07 |
| C2-C3 | π | C4-C5 | π* | 18.59 | 0.31 | 0.069 |
| C1-C6 | π | C9-C10 | π* | 16.32 | 0.29 | 0.062 |
| C15-C16 | π | C2-C3 | π* | 15.52 | 0.31 | 0.065 |
| C82-C83 | π | C22-C23 | π* | 13.92 | 0.29 | 0.057 |
| C35-C36 | π | C26-C27 | π* | 11.42 | 0.29 | 0.053 |
| C54-C56 | π | C61-C62 | π* | 9.26 | 0.32 | 0.051 |
| C54-C56 | π | C35-C36 | π* | 7.79 | 0.33 | 0.047 |
| C55-O57 | π | C59-C60 | π* | 4.15 | 0.43 | 0.041 |
| C55-O57 | π | C35-C36 | π* | 3.55 | 0.43 | 0.038 |
| C25-C31 | π | C25-C31 | π* | 1.5 | 0.29 | 0.019 |
| C35-H37 | σ | C26-S29 | σ* | 10.62 | 0.71 | 0.077 |
| C18-C20 | σ | C21-S30 | σ* | 8.32 | 0.92 | 0.078 |
| C26-C27 | σ | C31-C33 | σ* | 7.29 | 1.15 | 0.082 |
| C35-H37 | σ | C36-C55 | σ* | 6.77 | 0.99 | 0.074 |
| C26-S29 | σ | C14-C25 | σ* | 5.87 | 1.18 | 0.075 |
| C36-C54 | σ | C35-C36 | σ* | 5.5 | 1.29 | 0.075 |
| C82-C84 | σ | C22-C82 | σ* | 4.9 | 1.22 | 0.069 |
| C54-C56 | σ | C36-C54 | σ* | 4.28 | 1.26 | 0.066 |
| C78-H81 | σ | C2-O73 | σ* | 3.99 | 0.89 | 0.053 |
| C148-C149 | σ | C144-C149 | σ* | 3.47 | 1.31 | 0.061 |
| C23-C32 | σ | C20-C21 | σ* | 3.31 | 1.21 | 0.057 |
| C26-C35 | σ | C36-C54 | σ* | 3.09 | 1.24 | 0.055 |
| C60-C61 | σ | C54-C61 | σ* | 2.96 | 1.17 | 0.053 |
| C144-C145 | σ | C149-H154 | σ* | 2.69 | 1.13 | 0.049 |
| C89-N92 | σ | N92-C93 | σ* | 2.55 | 1.21 | 0.05 |
| C2-C3 | σ | C15-C16 | σ* | 2.19 | 1.3 | 0.048 |
| C34-C50 | σ | C32-C34 | σ* | 2.03 | 1.06 | 0.041 |
| C34-C50 | σ | C46-H49 | σ* | 1.65 | 1.01 | 0.037 |
| C32-C34 | σ | C46-H48 | σ* | 1.52 | 1.01 | 0.035 |
| C31-C33 | σ | C38-H39 | σ* | 1.46 | 1.02 | 0.035 |
| S11-C14 | σ | C12-C14 | σ* | 1.36 | 1.26 | 0.037 |
| C148-O155 | σ | C148-C149 | σ* | 1.17 | 1.51 | 0.038 |
| C26-C35 | σ | C35-H37 | σ* | 1 | 1.15 | 0.03 |
| C74-H75 | σ | C1-O72 | σ* | 0.67 | 0.89 | 0.022 |
| C22-S30 | σ | C32-C34 | σ* | 0.51 | 1.1 | 0.021 |
| N114 | LP(1) | C136-C137 | π* | 37.27 | 0.3 | 0.098 |
| N92 | LP(1) | C85-C89 | π* | 19.82 | 0.3 | 0.071 |
| O72 | LP(2) | C1-C6 | π* | 4.78 | 0.39 | 0.042 |
| O73 | LP(2) | C2-C3 | π* | 3.62 | 0.38 | 0.036 |
| O57 | LP(2) | C55-C60 | σ* | 21.41 | 0.76 | 0.115 |
| F67 | LP(2) | C58-C59 | σ* | 6.34 | 1.01 | 0.072 |
| O72 | LP(2) | C74-H77 | σ* | 4.5 | 0.73 | 0.052 |
| S30 | LP(1) | C21-C32 | σ* | 2.51 | 1.23 | 0.05 |

**Table S7:** Linear polarizabilities and major contributing tensors (*esu*) of the compounds **DOCR1** and **DOCD2**-**DOCD6**.

| **Compounds** | ***α_xx_* × 10^-22^** | ***α_yy_* × 10^-22^** | ***α_zz_* × 10^-22^** | **<α> × 10^-22^** |
| --- | --- | --- | --- | --- |
| **DOCR1** | 5.466 | 1.868 | 0.605 | 2.646 |
| **DOCD2** | 5.419 | 2.014 | 0.868 | 2.767 |
| **DOCD3** | 5.757 | 2.523 | 1.039 | 3.106 |
| **DOCD4** | 5.572 | 2.459 | 1.259 | 3.097 |
| **DOCD5** | 5.438 | 2.552 | 1.352 | 3.114 |
| **DOCD6** | 5.247 | 2.359 | 1.439 | 3.015 |

**Table S8:** Dipole moment (*u*) and major contributing tensors (*D*) of the studied compounds.

| **Compounds** | ***u*_x_** | ***u*_y_** | ***u*_z_** | ***u*_total_** |
| --- | --- | --- | --- | --- |
| **DOCR1** | -0.0003 | -3.254 | 0.00003 | 3.254 |
| **DOCD2** | -21.674 | -1.423 | 0.774 | 21.735 |
| **DOCD3** | 17.831 | -5.034 | 0.087 | 18.528 |
| **DOCD4** | 13.160 | -3.437 | 0.342 | 13.606 |
| **DOCD5** | -11.736 | -3.549 | -1.853 | 12.400 |
| **DOCD6** | -11.657 | -3.716 | -0.387 | 12.241 |

**Table S9:** The computed first hyperpolarizabilities (*β_total_)* and major contributing tensors (*esu*) of the studied compounds **DOCR1** and **DOCD2**-**DOCD6**.

| **Systems** | **DOCR1** | **DOCD2** | **DOCD3** | **DOCD4** | **DOCD5** | **DOCD6** |
| --- | --- | --- | --- | --- | --- | --- |
| ***β*_xxx_×10^-27^** | -0.000002 | -7.135 | 5.621 | 4.311 | -3.947 | -3.723 |
| ***β*_xxy_×10^-27^** | -0.522 | -0.284 | -0.423 | -0.415 | -0.420 | -0.364 |
| ***β*_xyy_×10^-27^** | 0.000001 | -0.049 | 0.058 | 0.020 | -0.010 | -0.002 |
| ***β*_yyy_×10^-27^** | 0.028 | 0.020 | 0.009 | -0.0003 | -0.004 | -0.003 |
| ***β*_xxz_×10^-27^** | -0.00001 | -0.008 | -0.019 | 0.077 | -0.073 | -0.054 |
| ***β*_yyz_×10^-27^** | -0.0000006 | -0.002 | 0.001 | 0.002 | -0.002 | -0.003 |
| ***β*_xzz_×10^-27^** | 0.0000004 | 0.005 | -0.003 | -0.005 | 0.004 | 0.007 |
| ***β*_yzz_×10^-27^** | 0.0004 | -0.0008 | 0.0006 | 0.004 | 0.0007 | -0.0001 |
| ***β*_zzz_×10^-27^** | 0.0000001 | -0.0002 | -0.0005 | 0.0004 | 0.0008 | -0.0007 |
| ***β*_total_ ×10^-27^** | 0.493 | 7.184 | 5.691 | 4.345 | 0.003 | 3.736 |

**Table S10:** Second hyperpolarizabilities and major contributing tensors (*esu*) of the studied compounds.

| **Compounds** | ***γ_x_*×10^-31^** | ***γ_y_*×10^-31^** | ***γ_z_*×10^-31^** | ***γ_total_*×10^-31^** |
| --- | --- | --- | --- | --- |
| **DOCR1** | 0.531 | 0.004 | 0.0001 | 0.535 |
| **DOCD2** | 1.667 | 0.009 | 0.0001 | 1.676 |
| **DOCD3** | 1.133 | 0.011 | 0.0001 | 1.144 |
| **DOCD4** | 0.704 | 0.007 | 0.0006 | 0.712 |
| **DOCD5** | 0.624 | 0.006 | 0.0005 | 0.631 |
| **DOCD6** | 0.582 | 0.005 | 0.0009 | 0.588 |

|  |  |
| --- | --- |
| **DOCR1** | **DOCD2** |
|  |  |
| **DOCD3** | **DOCD4** |
|  |  |
| **DOCD5** | **DOCD6** |


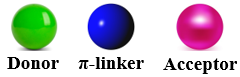


**Figure S1:** Structure of reference (**DOCR1**) and designed compounds (**DOCD2-DOCD6**).

| 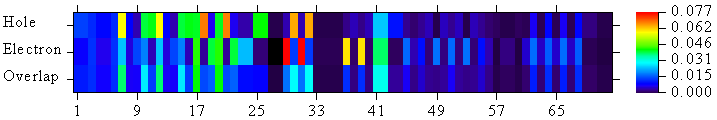 |
| --- |
| **DOCR1** |
| **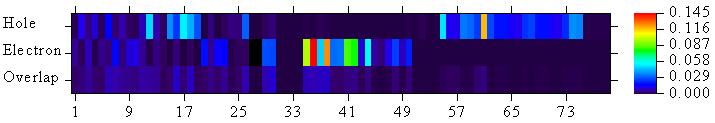** |
| **DOCD2** |
| **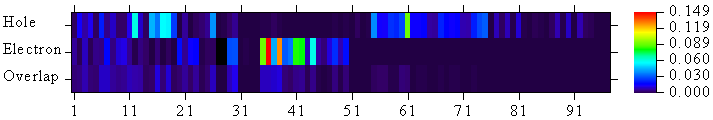** |
| **DOCD3** |
| **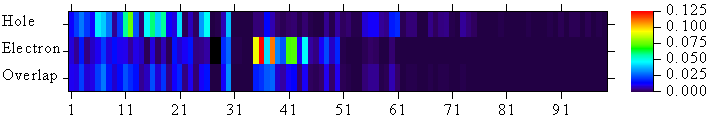** |
| **DOCD4** |
| **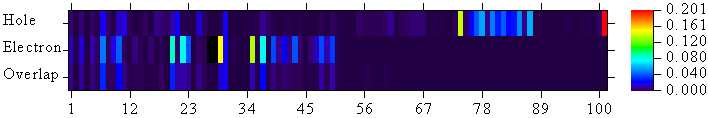** |
| **DOCD5** |
| **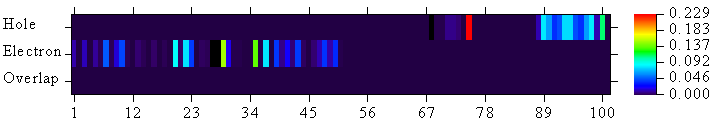** |
| **DOCD6** |

**Figure S2:** Pictographs of hole-electron transport analysis of **DOCR1** and **DOCD2-DOCD6.**

The IUPAC names of designed derivatives are listed as follows: (Z)-2-(2-((5-(4-(6-(4-(bis(4-(dimethylamino)phenyl)amino)phenyl)-4,4-dimethyl-4H-cyclopenta[1,2-b:5,4-b']dithiophen-2-yl)-2,3-dimethoxyphenyl)-4,4-dimethyl-4H-cyclopenta[1,2-b:5,4-b']dithiophen-2-yl)methylene)-5,6-difluoro-3-oxo-2,3-dihydro-1H-inden-1-ylidene)malononitrile (**DOCD2**); (Z)-2-(2-((5-(4-(6-(4-((4-(cyclopenta[b]indol-4(3H,4aH,8aH)-yl)phenyl)(4-(3,3a-dihydrocyclopenta[b]indol-4(4aH)-yl)phenyl)amino)phenyl)-4,4-dimethyl-4H-cyclopenta[1,2-b:5,4-b']dithiophen-2-yl)-2,3-dimethoxyphenyl)-4,4-dimethyl-4H-cyclopenta[1,2-b:5,4-b']dithiophen-2-yl)methylene)-5,6-difluoro-3-oxo-2,3-dihydro-1H-inden-1-ylidene)malononitrile (**DOCD3**); (Z)-2-(2-((5-(4-(6-(4-((4-(4aH-carbazol-9(4bH,8aH,9aH)-yl)phenyl)(4-(8aH-carbazol-9(9aH)-yl)phenyl)amino)phenyl)-4,4-dimethyl-4H-cyclopenta[1,2-b:5,4-b']dithiophen-2-yl)-2,3-dimethoxyphenyl)-4,4-dimethyl-4H-cyclopenta[1,2-b:5,4-b']dithiophen-2-yl)methylene)-5,6-difluoro-3-oxo-2,3-dihydro-1H-inden-1-ylidene)malononitrile (**DOCD4**); (Z)-2-(2-((5-(4-(6-(4-(bis(4-(10H-phenothiazin-10-yl)phenyl)amino)phenyl)-4,4-dimethyl-4H-cyclopenta[1,2-b:5,4-b']dithiophen-2-yl)-2,3-dimethoxyphenyl)-4,4-dimethyl-4H-cyclopenta[1,2-b:5,4-b']dithiophen-2-yl)methylene)-5,6-difluoro-3-oxo-2,3-dihydro-1H-inden-1-ylidene)malononitrile (**DOCD5**) and (Z)-2-(2-((5-(4-(6-(4-(bis(4-(10H-phenoxazin-10-yl)phenyl)amino)phenyl)-4,4-dimethyl-4H-cyclopenta[1,2-b:5,4-b']dithiophen-2-yl)-2,3-dimethoxyphenyl)-4,4-dimethyl-4H-cyclopenta[1,2-b:5,4-b']dithiophen-2-yl)methylene)-5,6-difluoro-3-oxo-2,3-dihydro-1H-inden-1-ylidene)malononitrile (**DOCD6**) .

| *IP* = *-E*_HOMO_ | (Equation S1) |
| --- | --- |
| *EA* = *-E*_LUMO_ | (Equation S2) |
| $X=\frac{\left[ IP+EA \right]}{2}$ | (Equation S3) |
| $\eta=\frac{\left[ IP-EA \right]}{2}$ | (Equation S4) |
| $\mu=\frac{E_{\mathrm{HOMO}}{+E}_{\mathrm{LUMO}}}{2}$ | (Equation S5) |
| $\sigma=\frac{1}{2\eta}$ | (Equation S6) |
| $\omega=\frac{\mu^{2}}{2\eta}$ | (Equation S7) |
